# Considerations for the selection and phenotyping of mouse models for the study of Alzheimer’s disease

**DOI:** 10.1016/j.xpro.2026.104633

**Published:** 2026-06-16

**Authors:** Sevda Boyanova, Loukia Katsouri, Julija Krupic, Kaitlyn Hair, Szu-Han Wang, Frances K. Wiseman

**Affiliations:** 1 UK Dementia Research Institute at University College London, UCL Queen Square Institute of Neurology, London WC1N 3BG, UK; 2 Sainsbury Wellcome Centre, University College London, London W1T 4JG, UK; 3 Evidence for Policy & Practice Information Centre, University College London Social Science Research Unit, University College London, London WC1H 0NU, UK; 4 Institute for Neuroscience and Cardiovascular Research, University of Edinburgh, Edinburgh EH16 4SB, UK

**Keywords:** Model Organisms, Neuroscience, Behavior, Special Issue, NMGN Focused Collection

## Abstract

All models are incomplete and must balance the need for experimental efficiency with the complexity of the natural world. To select and use models for the study of human disease, it is critical to understand what is of fundamental scientific importance and which limitations are thus of greatest concern. Here, we highlight key considerations for the design of research studies using mouse models of aspects of Alzheimer’s disease for both mechanistic and proof-of-principle intervention studies. This primer considers mouse model choice, including the strengths and limitations of genetically altered, pathological aggregates injection, and human iPSC-chimera systems. We also review key principles of experimental design, husbandry, and technical considerations for the phenotyping of clinically disease-relevant features, with a focus on behavior and cognition.

## Graphical abstract

**Figure:**
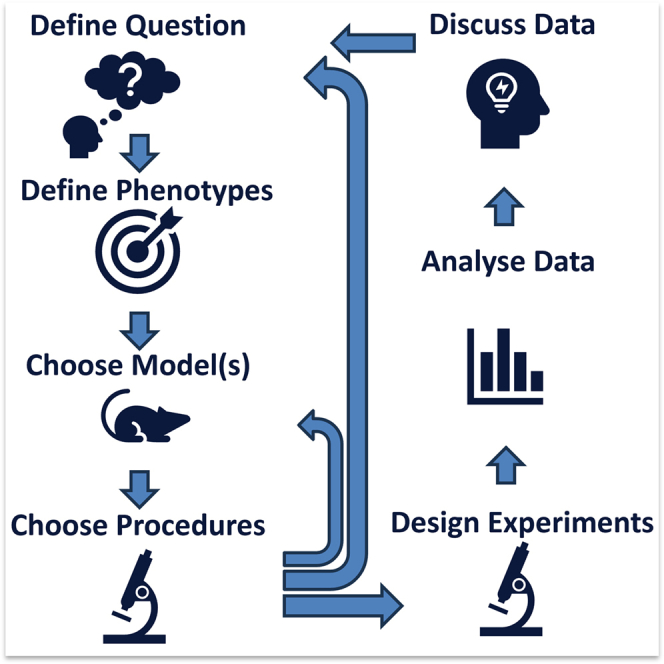

## Introduction

Alzheimer’s disease (AD) is the most common cause of dementia globally. In the UK around half a million people are living with an AD diagnosis, which is a major cause of mortality.^1^ The disease has two major forms, that share common underlying pathology and clinical features. Autosomal dominant AD (ADAD) is caused by mutations in the *APP*, *PSEN1* and *PSEN2* genes, whereas sporadic disease is caused by a combination of genetic and environmental factors, and typically has a later age of onset, and is called late onset AD (LOAD).^2^ Both forms of disease lead to the accumulation of aggregated amyloid-β plaques within the brain’s parenchyma, the accumulation of misfolded and hyperphosphorylated neurofibrillary tangles (NFTs) within neurons, and in the later stages, result in neuronal death and brain atrophy, particularly affecting the hippocampus and connected cortical regions.^3^^,^^4^

Neuropathological features take several decades to develop prior to the emergence of clinical dementia,^5^ which is characterized in the early stages by episodic short-term memory loss,^6^ decline in spatial memory and navigation^7^^,^^8^^,^^9^ as well as executive dysfunction,^10^ particularly inhibitory control and cognitive flexibility, including impaired judgment and planning. Later in clinical disease, additional cognitive domains are affected, including expressive and receptive language,^11^ and disinhibition. During end-stage, also known as advanced disease, motor domains are also affected, resulting in dyspraxia, dystonia, and Parkinsonism-like features.^12^ Before the onset of clinical disease, mild cognitive impairment (MCI) typically occurs; AD-associated MCI cognitive changes overlap with other types of MCI which have a differing biological cause.^13^ Many individuals experience new-onset anxiety disorder or depression, prior to a clinical AD diagnosis.^14^ In addition, depression, apathy, agitation, aggression, delusions and/or hallucinations occur in a high proportion of persons who have clinical AD.^15^^,^^16^^,^^17^ These behavioural and psychological dementia symptoms (BPDS) often result in a particularly high caregiver burden.

Genetic variation,^2^ particularly in the *APOE* gene, and life-history factors^18^ both contribute to LOAD risk. A number of treatments have been licensed for AD, including new anti-amyloid-β monoclonal antibodies, acetylcholinesterase inhibitors (e.g., Donepezil), and Memantine that targets glutamatergic transmission.^19^^,^^20^^,^^21^ Notably, current treatments either do not modify the course of disease or only partially slow the rate of cognitive decline, with unclear long-term benefit. These treatments also have significant side effects, some of which are life-threatening. Further medical research is thus required to develop safer and more effective treatments that halt the disease and/or facilitate recovery of brain health and function.

## Modeling Alzheimer’s disease using mice

Use of the domesticated house mouse (*Mus musculus*) in preclinical neuroscience research utilizes its practical features (small size, short generation time, relative tolerance of group housing and inbreeding), in addition to its relative genetic similarities to humans, and ease of genetic manipulation. Mouse models have been used to study various features of AD and play an important role in understanding the complex interactions between the brain and whole-body physiology, particularly at the key interfaces of the brain and the vascular, immune, and sensory systems. Moreover, *in vivo* systems are critical to our understanding of how molecular and cellular changes alter cognition and behaviour. Recent genetic and epidemiological studies have highlighted that immune, sensory and vascular health are key components of dementia risk,^18^ emphasizing the clinical importance of this biology. *In vivo* animal studies are required to mediate hypothesis testing and allow invasive observations of cellular functions within the context of an intact, interconnected brain that cannot be conducted using experimental medicine approaches because of ethical constraints.

In addition to the role in fundamental discovery research, *in vivo* studies are also required to test the efficacy and safety of newly developed AD therapies. For example, soon after the identification of causal ADAD genes, *APP* transgenic mouse models were developed that recapitulated key disease features, including accelerated amyloid-β accumulation, and associated physiological and cognitive changes.^22^ These early models were instrumental in the development of recently approved anti-amyloid-β antibody therapies.^23^^,^^24^^,^^25^ Moreover, *in vivo* preclinical work^26^^,^^27^ identified microhaemorrhage as a key side-effect risk of this treatment. Research using mouse models also identified the role of cerebral amyloid angiopathy, a subtype of AD pathology, in haemorrhage risk.^28^ This research was key to the development of new AD therapies and highlights the contribution of mouse models to improvements in the treatment of AD.

## Limitations of Alzheimer’s disease modeling in mice

Although around 85% of protein-coding genes are conserved between mice and humans, genetic conservation is not even across the genome, with higher rates of divergence occurring in immune (including microglial) and vascular genes.^29^ Human epidemiological,^18^ genetic,^30^ and multi-omics^31^^,^^32^ studies have highlighted the importance of both the neurovascular unit and microglial biology to AD, thus the genetic and functional conservation of these parts of the brain should be considered when evaluating the translational value of mouse research. The biology of key AD proteins differs between humans and mice. For example, differences in mouse APP processing reduce amyloid-β production,^33^ change amyloid-β aggregation kinetics,^34^ and developmental differences in tau splicing^35^^,^^36^ prevent the recapitulation of AD-associated shifts in tau isoforms in mice. Notably, although the introduction of ADAD causal mutations in *APP*, *PSEN1* and/or *PSEN2* into transgenic mouse models recapitulates the early stage of disease development well (accumulation of amyloid-β, and associated neuroinflammatory changes and dystrophic neurites), it does not elicit robust downstream alterations, including NFTs and neurodegeneration.^37^ Notably, this is not a limitation of neurodegenerative mouse models per se, as both prion^38^ and Huntington’s disease,^39^ can be recapitulated in mouse models, including all key aspects of disease, such as cell loss, brain atrophy and clinical changes. Differences in disease aetiology are likely to contribute to these differences. In particular, the complexity of the cascade of cellular and molecular events, including the amyloid to tau pathology transition that is central to AD may be challenging to model in mice without humanisation of multiple genes and/or cells.^40^

Moreover, the human brain is significantly more complex than the mouse, particularly in the upper cortical layers,^41^ due to differences in proliferation and differentiation of neurons, although conservation of fundamental circuits and cell-type roles occurs.^42^^,^^43^^,^^44^ The human brain has a higher proportion of white matter, largely because of the need for longer-distance connections due to its greater size^45^ and a higher proportion and diversity of GABAergic interneurons,^46^ although the excitatory/inhibitory balance is highly similar to mouse.^43^^,^^44^ Despite these anatomical differences, multiple cognitive functions are conserved between mice and humans, including associative learning, spatial navigation, attention, disinhibition and decision making. However, some aspects of complex human behaviour affected in AD cannot be readily studied in mice, including impairments in expressive and receptive language, the complete experience of human episodic memory, and delusions, hallucinations as well as the full complexity of other behavioural and psychological symptoms of dementia (BPSD), such as delusions or irritability. These molecular, cellular, structural and functional differences must all be considered during preclinical model selection and experimental planning.

## Practical guidance and considerations

### Define experimental questions and consider alternative research methods

**Figure 1:**
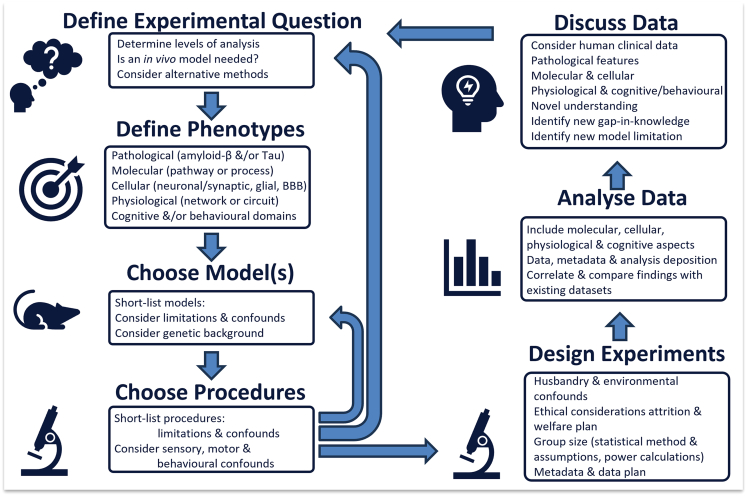
Flow chart for the selection and phenotyping of mouse models for the study of Alzheimer’s disease Define experimental question(s) and consider alternative research methods. Shortlist potential models taking account limitations and confounds, consider genetically altered models including transgenic and knock-in, complex genetic mouse models (spatial or temporal restricted), human cellular chimeric and pathogenic aggregate options and the effect of genetic background. Shortlist procedures for phenotypes of interest, consider potential sensory, motor and behavioural confounds, and known phenotypes of shortlisted models, iterating as required. Design experiments including identification of additional procedural, husbandry confounds and ethical and feasibility constraints. Conduct research and analyse, discuss, contextualise and deposit data.

A clear definition of the key hypothesis, and the expected phenotypes is required before model selection (Figure 1). This should include a definition of the relevant pathological, molecular, cellular, physiological and behavioural or cognitive features of diseases. In particular, understanding of how these features relate to one another should be considered. Use of the PREPARE framework^47^ and local review of study design can significantly enhance the quality of all animal work studies. This should include careful consideration of the 3Rs (replacement, reduction and refinement), and the potential harms and benefits of the planned work.^48^ New large human datasets, experimental medicine studies, complex human cellular modelling techniques, systematic preclinical data mining, and *ex vivo* studies can be used to replace a significant amount of *in vivo* research and enhance the design of any necessary animal work.

### Choosing a model: Consideration of limitations and confounds

If an *in vivo* study is required, careful consideration of models' strengths and limitations is necessary to match a model to a research question. A classical approach is to systematically consider construct, face and predictive validity of the model.^49^ For example, a researcher may want to study a mechanism related to amyloid-β pathology, synaptic loss and how this contributes to AD-associated episodic memory deficits. The model must robustly exhibit all relevant phenotype(s) (face validity). Furthermore, these phenotypes should develop with clinically relevant temporal and spatial (anatomical) sequence, in a model that has genetic/cellular relevance to disease (construct validity). Robustness, reliability (lack of variability between animals), and reproducibility (lack of variability between experiments) of phenotypes of interest is critical to model selection. Importantly if may be necessary to compromise between construct and face validity in some cases. Critically, predictive validity or translational value is the paramount consideration when selecting a model.

### Genetically altered models

Over 270 likely causal ADAD mutations have been identified; the majority of which are in the *PSEN1* gene, and the remainder are evenly spread between *APP* and *PSEN2*.^50^ Age of disease onset occurs on average earlier in *PSEN1* postcodon 200 mutation carriers and later in *PSEN2* mutation carriers. Mutation type is predictive of age of onset,^51^ and in ADAD and AD caused by Down syndrome (DSAD) genetic variation associated with LOAD risk (polygenetic risk score) had only a modest effect on disease.^52^ This evidence highlights that these genetic forms of disease are strongly deterministic. Mechanistically, these mutations either (i) cause the overproduction of *APP* gene products, including amyloid-β, (ii) alter the processing of APP to favour the generation of more amyloid-β^53^ or (iii) more aggregate-prone forms of the peptide.^51^ Notably, mutations in the tau gene *MAPT* do not cause AD but instead cause a type of Frontotemporal dementia (FTD), in which tau neuropathology that differs both biochemically and spatially from that of AD, develops in the absence of amyloid-β accumulation.^54^ This subtype of FTD shares some overlapping clinical and mechanistic features with AD but is a distinct clinical disease, thus in this review we will not focus on *MAPT* models of FTD. Numerous ADAD transgenic and gene-targeted animal models have been generated, including many that combine multiple ADAD mutations to elicit timely and robust neuropathology.^37^ Each of these models has a unique pattern of strengths and limitations, which must be considered when selecting a model for a given research project. Alzforum, curates a detailed database of these models.^55^ Here, we outline general principles to consider during model selection and use (Figure 1).

### Transgenic mouse models

The first widely used mouse models of aspects of AD were transgenics in which *APP* and/or *PSEN1* human minigenes carrying ADAD mutations, under the control of an artificial promoter, were randomly integrated into the mouse genome. Lines that developed robust amyloid-β pathology were selected for downstream use. The use of artificial promoters resulted in differences in spatial and temporal expression between lines and from endogenous APP. Moreover, transgenic human *APP* expression was typically elevated compared to endogenous mouse *App*, thus potentially affecting proteostasis and subcellular trafficking.^56^ Notably, a 50% elevation in *APP* gene products is sufficient to cause ADAD,^57^ illustrating the dosage sensitivity of APP.

Epigenetic effects on the *APP* transgene, resulting in parental inheritance modulation of APP expression, have been reported for the commonly used 5XFAD transgenic model.^58^ Disruption of endogenous mouse genes at the point of transgene insertion can affect neuronal biology in ways which confound AD-related phenotypes, as had been reported in the J20 transgenic model.^59^^,^^60^^,^^61^ Moreover, APP cleavage products, in addition to amyloid-β, are elevated in transgenic *APP* models, including the widely used 5xFAD and *APP*^*Swe*^*/PSEN1*^*dE9*^ lines. These include C-terminal fragments (APP-CTFs) that interact with cellular proteins, which when elevated cause changes to cell biology independent of the effects of amyloid-β.^62^^,^^63^ In some cases, these limitations can result in spurious findings,^64^ impeding translation. Furthermore, in transgenic models, the expression of mutant *APP* is limited to specific cell populations (often neurons) because of the use of artificial promoters, which does not reflect the wider endogenous pattern of *APP/App* expression. For example, a recent study showed that *App* expression in both neurons and oligodendrocytes contributes to amyloid-β pathology.^65^

### Knockin or gene-edited mouse models

The limitations of conventional transgenic mouse models led to the generation of knock-in or gene-edited models, in which the mouse genome was edited to humanize and/or introduce ADAD mutations into the mouse orthologues of *APP*, *PSEN1* and *MAPT*.^63^^,^^66^^,^^67^^,^^68^^,^^69^ To facilitate robust pathology development at endogenous gene expression levels, it is necessary to combine multiple ADAD mutations, as partial humanization of *App* in the mouse does not lead to the development of robust pathology, even in models that incorporate ADAD mutations.^67^ Each ADAD mutation causes distinct alterations to APP biology. For example, the commonly used K670N_M671L or Swedish mutation enhances the cleavage of APP by β-secretase,^70^ and changes the subcellular trafficking of APP.^71^ The E693G or Arctic mutation within the amyloid-β sequence, changes the aggregation of the peptide enhancing the formation of protofibrils^72^ and changing the structure of aggregates.^73^ Whereas the I716F or Iberian ADAD mutation, changes the interaction of APP C-terminal fragment with γ-secretase, favouring the production of longer aggregate-prone amyloid-β peptides greatly increasing the amyloid-β_42_/amyloid-β_40_ ratio.^63^ Combining multiple mutations results in biological perturbations that do not occur in ADAD, and results in amyloid-β secondary structures that are not found in human disease.^73^

Moreover, whether models based on ADAD disease mechanisms are representative of LOAD has been questioned. Alternative combinatorial approaches in which LOAD genetic risk loci are incorporated by genome editing into mice and then the resulting phenotypes are mapped using omics methods to stages and aspects of disease mechanism,^74^ can be used alongside existing ADAD models.

### Complex genetic mouse models (spatial or temporal restricted)

For some research questions, spatial and/or temporal restriction of pathology is critical. This can be mediated by either the use of Adeno-associated virus (AAV) mediated transduction, in which the route of administration, tropism of AAV capsid and choice of promoter are used to control when and where a transgene of interest is expressed. Alternatively, combinations of integrated transgenes can be used to mediate or stop expression of a gene of interest after administration of a small molecule, such as Cre-ER or tetracycline-transactivator (tTA) expression systems. These methods have been used to generate lines in which the expression of *APP/App* can be temporally and/or spatially controlled.^75^ When working with these complex systems, particular care regarding the selection of control groups is required, as both the genetic manipulation and the small molecule (e.g., doxycycline, tamoxifen) used to mediate gene expression, can also independently alter AD-relevant phenotypes.

### Human cellular, chimeric, and pathogenic aggregate mouse models

In addition to genetically altered models, chimeric mice engrafted with cells differentiated from human induced pluripotent stem cells (iPSC), are used to better understand the human-specific cellular response to AD pathology of a range of cell-types, including neurons, astrocytes and microglia.^76^^,^^77^^,^^78^^,^^79^ Long-term cellular engraftment of cells is facilitated by the use of recombination activating gene 2 (*Rag2*^−/−^) and/or interleukin 2 receptor gamma chain (*Il2rγ*^−/−^) lines, and for microglia human cell chimeric animals that also express human CSF1.^78^ In these models, *Rag2*^−/−^ and/or *Il2rγ*^−/−^ mediate immunosuppression, required for long-term engraftment, and hCSF1 is used specifically to promote human microglia growth and survival.^80^ These model systems are highly complex, and the proliferation, survival, and differentiation of human cells after injection can vary with each graft, and the type of cell transplanted. In some cases, this limits the manifestation and interpretation of whole-organism phenotypes, including behavioural changes. To understand defined fundamental molecular processes such as seeding and propagation of protein misfolding, injection of AD-associated amyloid-β and tau pathological aggregates into a specific brain region or ventricles of the mouse has been used.^81^ While these models are less commonly employed to study behaviour and cognition, several studies support the feasibility of using intra-cranial injection of soluble amyloid-β to understand affected memory processes or phenotypes.^82^^,^^83^^,^^84^^,^^85^ These two model systems – chimeric mice engrafted with iPSCs-derived human cells, and brain injection of disease-associated protein aggregates, can also be combined and have been particularly useful to elucidate the cellular and molecular responses to AD pathology.^86^

### Genetic background and sex

In addition to primary model choice, consideration of the genetic background of the mouse model is also necessary as this will impact of AD-relevant phenotypes.^87^ For example, the C57BL/6JOlaHsd genetic background carries a deletion of *Scna*^*-*^^88^ a Parkinson’s disease-associated gene, involved in neuronal function and neuron-glia interactions. Several mouse inbred genetic backgrounds, carry the mild retinal degeneration 8 (*Crb1*^*rd8*^) or the early-onset severe retinal degeneration 1 (*Pde6b*^*rd1*^) mutations, which cause retinal degeneration and blindness in affected mice.^89^ The *Crb1*^*rd8*^ mutation is found in many C57BL/6 N sub-strains^90^ and the more aggressive *Pde6b*^*rd*^ mutation in numerous strains, including uncorrected C3H and FVB/NJ backgrounds.^91^

Moreover, sex differences in mouse models of aspects of AD are an important consideration given the known effect of gender on disease.^92^ In several transgenic models, including, 5xFAD and *APP*^*Swe*^*/PS1*^*dE9*^ lines,^93^^,^^94^ female mice show more pronounced cognitive-behavioural phenotypes than males,^92^^,^^95^ for example, hyperactivity.^96^^,^^97^ Also, female mice have been reported to have heightened anxiety, and subtle deficits in object processing and social interaction which were not found in males.^95^ Similarly, in the *App*^*NL-G-F/NL-G-F*^ knock-in model, females show weaker discrimination between social and non-social olfactory stimulus compared to female wildtype mice, whereas males have normal social olfactory discrimination.^98^ This may relate to the higher levels of inflammatory markers, and/or amyloid pathology that occurs in female compared to male mice in these both transgenic and knock-in models.^92^^,^^93^^,^^94^^,^^99^^,^^100^

Thus, when selecting a genetically altered model for AD research the causal mutation, the nature of the genetic manipulation, the genetic background, the mode of inheritance, and the sex of the animals should be considered, as all can affect the translational relevance of the resultant biological discoveries.

## Behavioral phenotyping and cognitive testing approaches: Considerations and limitations

Understanding how AD pathological, molecular, and cellular alterations result in clinically relevant cognitive and behavioural changes is key to the translation of fundamental research to improvements in patient outcomes; particularly to mediate preclinical testing of the efficacy and safety of novel therapies. This requires a comprehensive understanding of both cognitive and behavioural alterations in preclinical models alongside key confounding factors. This can be ensured by efficient and robust quantification of motivation, strategy and performance across a range of cognitive and behavioural domains, complemented by systematic collection of relevant metadata.

**Figure 2:**
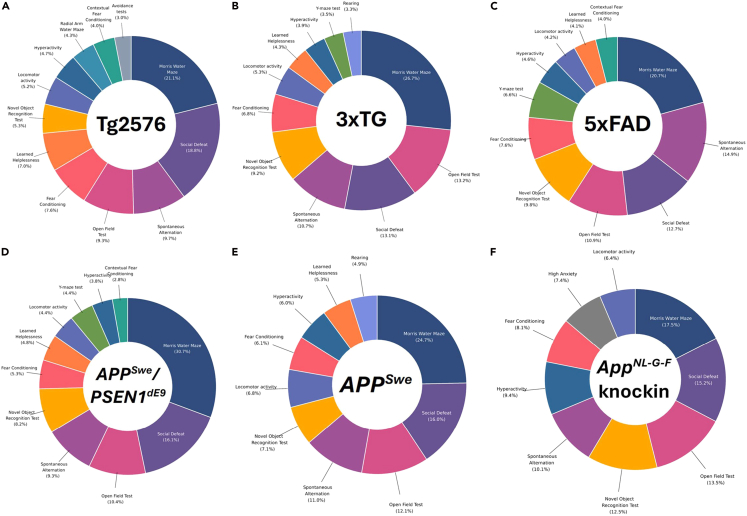
Donut plot of most frequently evaluated behavioural outcomes (estimated) in the five commonly used transgenic and the most commonly used knock-in mouse model derived from the AD-SOLES database (last update 18^th^ February 2026) Transgenic mouse models (A) Tg2576 (B) 3xTg, (c) 5xFAD, (D) *APP*^*Swe*^*/PSEN1*^*dE9*^, (E) *APP*^*Swe*^ and (F) knock-in gene targeted *App*^*Nl-G-F*^ mouse model. (A-D) Plots only shows outcomes where frequency was greater than n=100 articles, (E) plot only shows outcomes where frequency was greater than n=50 publication, (F) Plot only shows outcomes where frequency was greater than n=15 publications.

Systematic evidence evaluation tools, such as the living review AD-SOLES database can be used to inform model selection for a specific scientific question or phenotype. These tools are particularly useful to mine the extensive relevant mouse model behavioural literature to identify commonly used models for specific paradigms and link to the relevant underlying literature (Table 1 Figure 2).^101^ Here we summarise the relative proportion of behavioural/cognitive tasks for the top five most frequently reported transgenic models (Tg2576, 2565 articles; *APP*^*swe*^*/PSEN*^*1dE9*^, 2555 articles, 3xTG, 2339 articles; 5xFAD, 2113 articles; Generic *APP/PS1,* 1785 articles) and most frequently reported knock-in model, (*App*^*NL-G-F/NL-G-F*^, 241 articles). This analysis highlights the frequent use of the Morris water maze (MWM), social defeat and spontaneous alternation tasks (in T- or Y-mazes) in the literature, and the broad range of tasks used to study these models. Here we will consider these tasks and their strengths and limitations.

**Table 1:** Estimated number of scientific articles using animal models relevant to the study of behavioural and cognitive features of Alzheimer’s disease

	Common Model name	Genetic Mutations	MGI number	Number of articles
1	Tg2576	APP K670_M671delinsNL (Swedish)	4838841	2565
2	*APP*^*swe*^*/PSEN1*^*dE9*^	APP K670_M671delinsNL (Swedish), PSEN1: deltaE9	3524957	2555
3	3xTG	APP K670_M671delinsNL (Swedish), MAPT P301L, PSEN1 M146V	5003441	2339
4	5xFAD	APP K670_M671delinsNL (Swedish), APP I716V (Florida), APP V717I (London), PSEN1 M146L (A>C), PSEN1 L286V	3693208	2113
5	Generic *APP/PS1*	APP + PSEN1 (unknown mutations)	N/A	1785
6	*APP*^*Swe*^	APP K670_M671delinsNL (Swedish)	N/A	921
7	J20 (PDGF-*APP*^Sw,Ind^)	APP K670_M671delinsNL (Swedish), APP V717F (Indiana)	3057148	610
8	APP23	APP K670_M671delinsNL (Swedish)	2447146	484
9	TgCRND8	APP K670_M671delinsNL (Swedish), APP V717F (Indiana)	3589475	433
10	PDAPP(line109)	APP V717F (Indiana)	2151935	305
11	*App*^*NL-G-F*^Knock-in	APP K670_M671delinsNL (Swedish), APP I716F (Iberian), APP E693G (Arctic)	5637817	241
12	*APP*<V717I>	APP V717I (London)	3717572	203
13	*APP/PS1 –* transgene insertion 21	APP K670_M671delinsNL (Swedish), PSEN1 L166P	3765351	191
14	mThy1-hAPP751 (TASD41)	APP K670_M671delinsNL (Swedish), APP V717I (London)	5491622	169
15	*APOE4* Knock-In generic	Replacement of *Apoe* with human *APOE4* allele	N/A	162
16	*APOE* Knock-out generic	Loss of function of *Apoe*	N/A	159
17	*APP* Knock-out generic	Loss of function of *App*	N/A	137
18	PS/APP	APP K670_M671delinsNL (Swedish), PSEN1 M146L (A>C)	N/A	126
19	Ts65Dn	Translocation chromosome, Mmu 17^16^, 13.4 Mb of distal chromosome 16 fused with ∼10 Mb of chromosome 17 proximal to the centromere	2178111	121
20	Tg-SwDI (APP-Swedish,Dutch, Iowa)	APP K670_M671delinsNL (Swedish), APP E693Q (Dutch), APP D694N (Iowa)	3721992	117
21	*APP* Knock-in (ADF)	APP K670_M671delinsNL (Swedish), APP V717I (London), APP E693Q (Dutch)	3783884	112
22	*APOE4* Targeted Replacement (*Apoe*^*tm3(APOE*^*∗*^*4)Mae*^)	Replacement of *Apoe* with human APOE C130R (ApoE4)	N/A	106
23	*APOE3* Knock-In	Replacement of *Apoe* with human *APOE3* allele	N/A	88
24	PS1 A246E	PSEN1 A246E	N/A	62
25	PS2APP	APP K670_M671delinsNL (Swedish), PSEN2 N141I (Volga)	4412048	62
26	APOE2 Knock-In	Replacement of *Apoe* with human *APOE2* allele	N/A	60
27	TgAPP-SL	APP K670_M671delinsNL (Swedish), APP V717I (London),	5004645	59
28	Tg-ArcSwe	APP K670_M671delinsNL (Swedish), APP E693G (Arctic)	4420229	56
29	TASTPM (TAS10 x TPM)	APP K670_M671delinsNL (Swedish), PSEN1 M146V	N/A	53
30	APP NL-F Knock-in	APP K670_M671delinsNL (Swedish), APP I716F (Iberian)	5637816	52
31	E4FAD (5xFAD cross)	APP K670_M671delinsNL (Swedish), APP I716V (Florida), APP V717I (London), PSEN1 M146L (A>C), PSEN1 L286V	N/A	50
32	E3FAD (5xFAD cross)	APP K670_M671delinsNL (Swedish), APP I716V (Florida), APP V717I (London), PSEN1 M146L (A>C), PSEN1 L286V	N/A	40
33	PS cDKO	Loss of function *PSEN1* and *PSEN2*	N/A	37
34	APP(V717I) x PS1(A246E)	APP V717I (London), PSEN1 A246E	N/A	36
35	APP751SL/PS1 KI	APP K670_M671delinsNL (Swedish), APP V717I (London), PSEN1 M233T, PSEN1 L235P	N/A	36
36	Tg4-42	Model producing Aβ_4-42_ fragments	–	33
37	BACE1 Knock-out	Loss function of *Bace1*	3603141	32
38	APOE3 Targeted Replacement	Replacement of *Apoe* with human *APOE3* allele	N/A	23
39	AD11	Express a recombinant monoclonal antibody αD11 targeting NGF	N/A	22
40	APPDutch	APP E693Q (Dutch)	4438890	19
41	PSEN1(M146V) Knock-In	PSEN1 M146V	1930937	17
42	PSEN2 Knock-out	Loss of function of PSEN2	N/A	17
43	Tg2576/Tau(P301L) (APPSwe-Tau)	APP K670_M671delinsNL (Swedish), MAPT P301L	N/A	17
44	PS1 P264L	PSEN1 P264L	3608968	16
45	APP E693Δ-Tg (Osaka)	APP E693del (Osaka)	N/A	15
46	APP-YAC	Human APP construct from yeast artificial chromosome	2447591	15
47	PLB4 (hBACE1)	Human BACE1 construct	N/A	14
48	PSEN1 Knock-out	Loss of function of PSEN1	N/A	14
49	GFAP-apoE4	Expression of human APOE4 under human GFAP promotor	3057182	13
50	APPSw-NSE	APP K670_M671delinsNL (Swedish)	N/A	12

Estimated number of scientific articles including behavioural or cognitive data, listed by the top 50 most frequently used models generated from the AD-SOLES database (18^th^ February 2026). Introduced genetic alteration including ADAD causal mutation and Mouse Genome Informatics (MGI) reference number where available. In several cases clear attribution of reported outcomes to a specific line was not possible, and/or a models MGI was not available (N/A). The original list of models used for AD-SOLES was derived from AlzForum, with additional models collated from reviews of the literature.

### Memory deficits: Considerations and limitations

Numerous subtypes of memory can be tested in mouse models of AD. Spatial short and long-term memory is classically tested using Y-maze,^102^ T-maze,^103^ and Barnes maze^104^ paradigms. In these tasks animals are motivated by novelty, food/water-reward or to escape an adverse environment. Spontaneous alternation tasks in Y- or T-mazes largely rely on exploratory drive and recognition of a novel environment. Historically, the MWM^105^ was often used to test spatial memory in mice; in this task first developed for rats, animals were trained to find an escape platform in a pool of opaque coloured water. However, studies have highlighted that the welfare burden of swimming tests is very high in mice,^106^ and the induction of stress during MWM testing has been reported to impair learning.^107^ This may confound the translational relevance of this task to neurological disease, such that less stressful paradigms should be considered and prioritized. Notably, in all spatial memory tasks, animals may also use non-spatial strategies, thus careful assessment is required to determine if this occurs. Using virtual reality versions of spatial tasks, can address this limitation, allowing both the manipulation of testing conditions to probe potential non-spatial strategy use, precise control over the perceptual experience during the test, removal of non-visual stimuli, and the acquisition of considerable control data during testing.^108^^,^^109^

Object and social short and long-term memory can also be tested in mice, using the novel object recognition task (NOR)^110^ and the memory variant of the Crawley three-chamber task.^111^ Key controls for the NOR include ensuring animals have successfully habituated to the testing arena prior to object presentation, that the selected objects hold similar levels of interest to the animals, and that both the position and object identity are randomised (and/or blocked by primary variable of interest). Social memory tasks may be confounded by underlying changes in social preference, such that some genotypes of animals may have impaired social interest to non-familiar mice, such a phenotype has been reported in *APP*^*Swe*^*/PS1*^*A246E*^ mice compared to wildtype animals.^112^

A key and early aspect of AD clinical development is a decline in episodic memory, the recollection of previous events, including key integrations of *when* and *where* they occurred and in *what* context. Aspects of episodic memory can be tested in *what-where-when* tasks in people, but it has been highly challenging to formulate mouse tasks that demonstrate integration of *what-where-when* information,^113^ which is central to the concept of episodic memory. However, key aspects of episodic memory can be measured in mice. This includes the object location task,^110^ a *what-where* variant of NOR. In this version of the task, an animal is tested to determine if it can preferentially recall both the nature of an object and its position in space. *What-where* learning can also be tested using paired-associated learning (PAL) tasks, either in touchscreen^114^ or operant chamber platforms. Notably, conceptually similar PAL task in the Cambridge Neuropsychological Test Automated Battery (CANTAB) task battery, also shows early deficits in people who have AD and MCI.^115^ In some studies these deficits have been linked with biomarkers of the accumulation of amyloid-β,^116^^,^^117^ indicating the disease relevance of the PAL deficits in ADAD genetically altered mouse models. In the touchscreen variant of these tasks, animals are trained to recognize the form and position of an image.^114^ As in object memory tasks, pairs of images that hold similar interest to mice, and have similar levels of visual difference, are critical to the running of this task. Adding contextual clues to the testing arena (such as distinctive arena floor patterns) and/or running tasks at varying time-periods, has been added to further increase the episodic nature of the task,^118^ however it is still much debated if these fully recapitulate human episodic memory. Recently, subtle impairment in cognitive processes before evident memory impairment is observed in an appetitive delayed matching-to-place task in an event arena,^119^ this may reflect processes similar to AD-related MCI.

### Executive dysfunction: Considerations and limitations

Executive function is the overarching term for several processes that facilitate complex often goal-oriented outcomes. These behaviours are often impaired in people who have neurodegenerative diseases, including AD.^120^ Contributing processes include working memory, attention, cognitive and emotional inhibition and cognitive flexibility. Attention and cognitive inhibition can be measured in mice using the 5-choice serial reaction task, in which an animal is rewarded for responding to a specific cue and not rewarded for responding prematurely or incorrectly. Deficits in this task have been reported in amyloid-β accumulation models.^121^^,^^122^ Cognitive flexibility can be tested in tasks such as delayed alteration T-maze or Y-maze. A combination of attention and cognitive flexibility is tested in attentional-set-shifting tasks such as the Wisconsin Card Sorting Test (WCST) or the Intra-Extra Dimensional Set Shift task (IED) in the CANTAB test battery, which are impaired in persons with AD. In these tasks, after learning a simple rule, participants must switch to a new rule and attend to a previously disregarded stimulus. In mice, a conceptually similar touch-screen task has been developed (visual discrimination and reversal) in which animals first learn to discriminate between two simple visual cues, then these are reversed, and the gradient of re-learning is assessed.^123^ Deficits in this task have been reported in amyloid-β models in some but not all studies.^121^^,^^124^^,^^125^^,^^126^^,^^127^^,^^128^ Animals are typically motivated to complete this task by the restriction of food or water. Differences between genotypes in the response to food or water restriction may occur, which may impact behavioural or cognitive performance. Moreover, particular care should be taken during experimental design to account for the effects of caloric restriction and diet changes necessary for task motivation on pathology development. Providing free access to water containing 2% citric acid, as an alternative to water restriction, to motivate mice can be considered for water-rewarded decision making task^129^ but the efficacy of this refinement should be tested for each paradigm and strain.^130^ Cognitive flexibility can also be studied in other learning paradigms, including the Barnes and MWM reversal tests. In all cases, care must be taken to consider whether learning or experience will be fixed between study groups prior to the reversal or shift of the learning goal, as the initial learning rate may also be impaired in these tasks.

### BPSD features: Considerations and limitations

Changes to emotional state and related behavioural and psychological changes, such as depression, anxiety apathy and aggression are a common feature before and after the development of AD cognitive changes.^14^^,^^15^^,^^16^^,^^17^ Despite the very high impact on caregivers^131^ and persons with dementia, these features of the disease are relatively understudied. These aspects of disease are also frequently recapitulated in mouse models of disease.^132^ In addition to being key clinically relevant behavioural outcomes, these changes can also impact on task engagement and completion, affecting the measurement of other cognitive domains. Thus, all researchers undertaking cognitive testing should also consider the impact of these phenotypes during behavioural testing.

Classically, anxiety is measured in the elevated plus and elevated-zero mazes, light-dark box and aversive open field. These tasks rely on measuring the balance between drive to explore a novel environment and avoidance of potentially dangerous environments.^133^ Particular care regarding the previous experience of the animals being tested is required for these tasks because of the effect of experience on anxiety. Moreover, anxiety testing can elevate stress beyond the time in the test, impacting performance in subsequent tasks. Apathy, defined as a decline in motivation, can be studied in mice using the progressive ratio task, in which an increasing level of effort is required to achieve a reward.^134^ Mixed results have been observed in this task in mouse models of amyloid accumulation.^135^ Anhedonia, a reduction in preference for pleasurable experiences, is more frequently reported in persons who have AD.^136^^,^^137^ In mice, anhedonia is quantified using the sucrose preference test.^138^ Social withdrawal occurs in many people who have AD, complex social and neurobiological aspects of the disease likely underlie this important clinical feature. Some aspects of this may be modelled in mice, in which social preference can be tested using the Crawley three-chamber task (social motivation phase). Increased aggression is observed in around 40% of AD patients and often is concurrent with the development of delusions.^139^ Aggression can be quantified in mice using the resident intruder or social defeat test, but whether this reflects the complex manifestation of aggression in a person with AD is uncertain. For both tests, particular care must be taken regarding the identity (sex, genotype, genetic background) and prior experience of the non-specific mouse used to elicit a response. Previous social experiences, including those during early life and at the time of testing, may impact performance in these tasks. Therefore, consistency in breeding and maintenance is particularly important to reduce confounds in these tasks. Agitation, particularly at certain times of day, is frequently experienced by AD patients. Elevated activity levels, in some cases during particular phases of the circadian rhythm, are observed in a number of amyloid-β accumulation models.^140^^,^^141^^,^^142^^,^^143^^,^^144^^,^^145^ Further research is needed to understand if this has a similar neurobiological underpinning to the agitation experienced by AD patients. The testing of depression in mice is controversial, in part because of the complexity of this phenotype in humans, with the validity of the commonly used forced swim test having been questioned.^146^ In addition to these specific tests, performance in the marble burying, burrowing and nest building tasks,^147^^,^^148^ which represent typical daily behaviours in rodents, has been linked with both apathy and anxiety, deficits in these tasks have been reported in amyloid-β accumulation mouse models.^149^^,^^150^^,^^151^^,^^152^

### In-cage behaviors: Considerations and limitations

A number of platforms have been developed to quantify in-cage behavior,^153^ including systems designed to assess specific behaviours or activities, such as motor activity and climbing,^154^^,^^155^ working and object memory,^156^ and spatial learning and reversal.^157^ In some systems, water access is used to motivate animals to participate in specific learning and cognitive tests, care must be taken to monitor and control for genotype differences in motivation and learning, which may result in potential dehydration and welfare impacts.^158^ These systems have been particularly used to study circadian rhythm and sleep disturbances in mouse models of aspects of AD, contributing significantly to the mechanistic understanding of these clinically important aspects of disease.^159^ In addition, in-cage testing platforms have been used to demonstrate differences in impulsivity, cognitive flexibility and attention,^99^^,^^160^^,^^161^ object and spatial learning.^99^^,^^162^ Behavioural monitoring in first-generation systems was mediated by radiofrequency identification (RFID) tagging of animals to facilitate individual identification in group-housed environments, and/or infrared beam grids in which beam breaks are used to track movement. The resultant data is then typically analysed by supervised methods to quantify behavioural outputs of interest, in commercial platforms, this software is often proprietary, impeding full data transparency.^163^

The development of open-access pose estimation annotation tools (e.g., DeepLabCut^164^ and SLEAP^165^) and supervised classification software such as Simple Behavioural Analysis (SimBA),^166^^,^^167^ enables researchers with limited computational experience to develop their own pipelines for the quantification of in-cage behaviours, as well as performance in classical tasks. These supervised methods can be used to test defined hypotheses that are embedded in current understanding of behaviour. Unsupervised methods such as VAME^168^ and MoSeq,^169^^,^^170^ can be used to discover and define new behaviours, and elucidate subtle differences between mouse models and controls not detected using classical methods. Typically, these methods have higher computational demands and require considerable mathematical and coding expertise to implement in comparison to supervised methods. Moreover, to date unsupervised methods have been principally applied to temporally short recordings in low complexity environments (such as a simple open field).^168^^,^^169^^,^^170^ However, they can be particularly powerful to quantify complex and subtle behavioural differences in mouse models of amyloid-β accumulation.^168^^,^^171^ Addressing the limitations of current methods, including lowering the amount of information required for training datasets, improving computational efficiency, developing methods for occluded areas, and improving 3D and group methods, will increase the value of these tools, and thus the value of in-cage phenotyping to AD preclinical research. Similarly, open sharing of model training methodology, for example, features used to train behavioural classifiers, allows standardisation of future data collection and analysis, enhancing reproducibility.

### Potential sensory and motor confounds of behavioral testing

All cognitive and behavioural tests rely on sensory input and motor function, and AD-relevant pathomechanisms can impact both. These features of disease may be recapitulated in mouse models or may be perturbed due to non-AD relevant mechanisms. Therefore, sensory and motor function should be considered when interpreting phenotyping outcomes. Many tests rely on visual inputs. *App* is expressed in retinal ganglion cells, and amyloid-β accumulates in the retina,^172^ triggering an inflammatory response in the *App*^*NL-G-F*^ KI model.^173^ Assessment of visual acuity in this line has not detected any genotype-specific deficits,^172^ but care should be taken to confirm unimpaired visual function in these models. Other tasks may rely on auditory and olfactory cues, thus testing these functions may be important in some cases. Motor function impairments and hyperactivity have been reported in some *APP* transgenic models,^174^^,^^175^ and these may impact task performance in tests particularly those with high motoric demands, such as operant or swimming tasks.

### Cellular and functional *in vivo* electrophysiology: Considerations and limitations

To understand the neurophysiological underpinnings of AD-relevant behavioural changes *in vivo*, several methods have been used, including single-units, local field potential (LFP) and electroencephalogram (EEG) electrophysiological recordings in the hippocampus and the entorhinal cortices.^176^^,^^177^^,^^178^^,^^179^^,^^180^^,^^181^^,^^182^^,^^183^^,^^184^^,^^185^^,^^186^^,^^187^^,^^188^^,^^189^ These data can be correlated with performance in several tasks, such as a simple^190^ and forced-choice^177^ T-maze alternation task, Morris Water maze,^186^^,^^190^^,^^191^^,^^192^^,^^193^^,^^194^ radial arm maze,^194^ spatial recognition memory,^178^ novel object recognition,^195^ elevated plus maze,^186^ operant conditioning task,^178^ contextual fear conditioning (CFC),^183^ and path integration.^189^ Most of these tests probe spatial and object memory, and/or navigational abilities, which are key functions of the hippocampal-parahippocampal areas,^196^^,^^197^ affected early in AD. Cortical LFP/EEG, in both the medial prefrontal^176^ and parietal^192^ regions have been used to link network changes with memory consolidation. This method is comparable with EEG recordings in people, and is useful to understand disease-related changes in neural circuit function, mediated by amyloid-β pathology, such as cell hyperactivity.^198^^,^^199^

*In vivo* electrophysiology has been used to show that mice expressing mutant human tau in the entorhinal cortex show reduced grid cell firing and periodicity^190^; and neuronal activities are implicated in tau pathogenesis.^200^ Similarly, these methods have linked amyloid-β^201^ and Tau pathology^202^ with a decrease in burstiness and firing rate when an animal is at rest. Reported changes in excitatory neurons also include reduced spatial information and stability, and an increase in field size in hippocampal place cells in most^177^^,^^178^^,^^184^^,^^194^^,^^203^^,^^204^ but not all reports,^183^ and changes in the gridness score, spatial information, and cell numbers of medial entorhinal grid cells.^189^ Cell connectivity and rhythmicity,^188^^,^^189^ and changed inhibitory cell properties, especially of parvalbumin positive interneurons^193^^,^^205^ have been elucidated in mouse models of aspects of AD, providing key new understanding of cellular disease mechanisms. These changes are likely linked to the reported aberrations in LFP network properties in delta (0.5-4 Hz), theta (6-11 Hz) and gamma (30-150 Hz) frequency bands, as well as sharp-wave ripples.^176^^,^^178^^,^^179^^,^^180^^,^^181^^,^^184^^,^^188^^,^^206^ In general, changes in network and cellular firing properties crucially depend on the mouse’s behavioural state, e.g., rest versus running^202^ or exploring,^193^ or running versus sleep.^176^ This research has been critical to our understanding of how AD neuropathology results from and causes changes in brain function.

Despite some progress in this field, establishing a direct link between cognitive impairments and electrophysiological properties remains challenging due to immense technical difficulties in performing electrophysiological recordings in complex tasks in aged/or impaired mice. As a result, behavioural testing and neural recordings are often done in separate mouse cohorts, matched by age and genotype. For example, Ying and colleagues^189^ recorded from the medial entorhinal cells and showed that grid cells,^207^ which fire in symmetrically arranged fields, show reduced spatial information in the J20 transgenic mouse model of amyloid-β pathology compared to controls, and are more affected than other functional cell types. In parallel, in an independent experiment, they demonstrated that this model was impaired on the path integration task, commonly viewed as depending on grid cell function.^208^^,^^209^ In another example, Jun and colleagues^183^ showed that *App*^*NL-G-F*^ mice exhibited a deficit in the contextual fear conditioning paradigm. Using a different cohort of mice, they found a deficit in place cell remapping, as measured on the linear tracks. This research links impairments in remapping, differentiation between different contexts and impairments in associate-memory.

Alternatively, the cognitive deficits are first established on a task(s) of choice^190^ and then neural correlates of cognitive performance are identified in a separate, simpler experiment (e.g., in an open field). This affords greater granularity in establishing potential causal relationships between the measured physiological properties and cognitive impairments. Following this approach, Cacucci and colleagues found that in the aged Tg2576 model of amyloid-β pathology, spatial information and field size in hippocampal CA1 place cells, characterized in a square open enclosure, negatively correlated with spatial working memory, assessed in a T-maze forced-choice alternation task.^177^ Together, this provides insight into the cellular mechanisms that may underlie this key cognitive feature of AD. In addition to the approach of studying behaviour and cellular functions in aligned but independent experiments, combined studies of behaviour and electrophysiology have been undertaken, directly correlating neural activity with complex^178^ or simpler running behaviours.^206^ This more detailed approach is particularly valuable and informative, providing unique insight into the cellular mechanisms that result in AD-relevant cognitive changes.

Despite clear benefits, combining behavioural measurements with electrophysiological recordings also entails important additional limitations. First, it adds a significant complexity related to execution of the experiment compared to behavioural testing alone. Secondly, the direct comparison of disease-related neural signatures between mouse models and humans must be applied with care. Ideally, multiple outcome measures in different experimental frameworks should be compared when possible. Furthermore, some monitoring techniques are invasive, damaging the blood-brain barrier and/or the brain, potentially introducing confounds. Minimally invasive methods, such as two-proton imaging, which require only the insertion of a cranial window, can be used to limit this potential issue. In addition, the scope of recording is limited to the implanted area, which must be considered when interpreting the results. Thus, particular care should be taken regarding neuroanatomical and data reporting accessibility and transparency.^210^ New recording techniques such as Neuropixels^211^ are particularly beneficial for allowing simultaneous recordings of hundreds of cells from multiple brain regions. Finally, technical challenges related to the processing of large and complex datasets (e.g., spike sorting) must be considered, particularly when movement artefacts and implant drift are present and recordings are made over longer periods. Standardized data analysis toolboxes such as Kilosort for spike sorting^212^ are particularly useful to ensure the data quality.

### Identification and evaluation of procedural and husbandry confounds

Once the model, behavioural, cognitive or physiological procedures have been determined potential procedural and husbandry confounds should be evaluated. Care should be taken regarding the source of the control animals (wildtypes from littermates or from different colonies or suppliers) as genetic and environmental differences between colonies and suppliers can contribute significantly to phenotypes in mice. Wildtype performance affects phenotypic effect sizes in mouse models of aspects of AD.^213^ Consideration of animal facility conditions, particularly the day-night cycle and lighting, and how this interacts with the natural circadian rhythm of mice can further refine data collection. Husbandry conditions during breeding and early development, and genetic differences between colonies can affect phenotypes throughout life. Thus, sourcing controls and genetically altered animals from the same facility and ideally the same colony is imperative to ensure the validity of resultant data. Enhancing husbandry practices to improve welfare, such as caging enrichment,^214^^,^^215^ and changes to handling^216^ can reduce animal stress, enhance learning, while maintaining research reproducibility.^217^ Handling amounts may contribute to the effect sizes of AD mouse phenotypes.^213^ Notably, social isolation accelerates amyloid-β pathology development in numerous models,^218^ thus consideration of the management of lone-housing is required during experimental planning.

Dietary modifications and caloric restriction also impact amyloid-β pathology development in mice^219^^,^^220^^,^^221^^,^^222^^,^^223^; therefore, care should be taken when using food restriction and reward paradigms for cognitive phenotyping. Furthermore, touch-screen tested male WT and *APP*^*Swe*^*/PS1*^*d9E*^ mice were found to perform better in the Morris water maze compared to behaviourally naïve and non-calorie restricted littermates,^224^ suggesting that the touchscreen training can improve cognitive performance and induce phenotypic shifts. Type and duration of anaesthesia required for any procedures should be considered, and changes to AD-relevant phenotypes have been reported for some model-anaesthesia combinations.^225^

### Ethical and feasibility constraints

Feasibility of the proposed model, study timepoints and procedures should then be considered. Elevated attrition rates and phenotypes impacting on animal welfare have been reported in several models relevant to the study of AD. This includes elevated mortality in J20,^226^ *APP*^*Swe*^*/PS1*^*d9E*^^227^ transgenic lines attributed to spontaneous seizures, and hyperactivity and stereotypic behaviours reported in the CRND8 transgenic line.^145^ These welfare impacts may preclude the use of some models or require a significant increase in the number of animals needed for a study. Also, the number of animals that might require euthanasia due to adverse welfare event prior to the study completion should be taken into account as this could make a planned timepoint or procedure is unfeasible. Reduction strategies, including modified breeding schemes and application of advanced statistical methods, such as linear mixed modelling^228^ and Bayesian approaches,^229^ can be used to reduce the number of animals needed to power a study enhancing feasibility in some cases. To enable this, primary data for the phenotypes of interest should be obtained either by pilot studies or from trusted sources so that robustness, reliability and reproducibility of key phenotypes can be assessed and feasibility of the study determined. Integrated robust phenotyping datasets generated in well-controlled studies such as those at MODEL-AD,^230^ and systematic data-mining platforms^101^ facilitate this, offering researchers unprecedented opportunities to consider all available evidence to guide model selection for their studies. In addition to the role of these data in model selection, they can also be used for power calculations, in statistical software or online-tools^231^^,^^232^ to determine the numbers of animals needed for given study. Identification and elimination of causes of experimental variation can further reduce the number of animals needed for a particular research question.

### Analyse, discuss, contextualize, and deposit data

The analysis of animal research data not only tests a specific hypothesis and generates novel understanding of AD mechanisms or the potential of an intervention or treatment but also enables improved model choice and identifies areas for new model development for others. This is enabled by study outputs that fully comply with ARRIVE 2.0^233^ and FAIR principles^234^^,^^235^ ensuring that they can be incorporated into future large language model mediated meta-analysis,^101^ significantly enhancing the translational value of individual research outputs. Large animal behavioural data repositories such as MOUSEBYTES,^124^ a database for touch-screen based behavioural assays, in which data is systematically quality controlled, using a consistent data dictionary, with complete metadata recorded in a machine-readable format, can significantly facilitate meta-analysis of preclinical research, enhancing the value of the research conducted.

## Conclusion

Recent clinical advances in therapies for the treatment of AD,^20^^,^^21^ illustrate both the tractability of disease-modifying therapy and the importance of *in vivo* research for the development of effective treatments.^26^^,^^27^^,^^28^ Here, we outline the key considerations for high-quality *in vivo* behavioural, cognitive and electrophysiological fundamental research to further understanding of AD-relevant mechanisms to facilitate the development and testing of next-generation disease treatments. As with all preclinical research studies, adherence to principles of experimental design and reporting, such as ARRIVE 2.0,^233^ are critical to the translational value of research. Full and accurate reporting mediates systematic analysis of research evidence to determine the robustness and reproducibility of a specific mechanism and/or novel treatment. In particular, for AD preclinical research, attention to the composition of experimental groups (age, sex, and genetic origin), relevant metadata, potential confounds and the rationale for the model or models selected for study is required. Notably, no one AD preclinical model recapitulates all aspects of human disease, and thus no one model is suitable for all research questions. Similarly, no one task or method is currently available to capture the full extent of all AD-relevant behavioural and cognitive changes. Therefore, a battery of complementary tasks is typically required for both mechanistic and proof-of-principle intervention studies. During task and model selection and development, downstream applications of the research, including compatibility with invention studies, including drug dosing routes and frequencies, are also important to ensure the long-term value and impact of fundamental research. This may be particularly relevant to the use and development of in-home cage assessment methods. Careful consideration should be given to how to determine the relative efficacy of a treatment and/or the magnitude of a specific mechanism in comparison to other potential effects; transparent and standardized research methods and data formats can facilitate this. Lastly, the identification and reporting of likely potential safety impacts and/or confounds will ensure that all *in vivo* research provides the greatest insight possible and will mediate rapid improvements in AD treatments and outcomes for the millions of persons globally affected by this debilitating disease.

## Acknowledgments

F.K.W. and S.B. are supported by the 10.13039/501100017510UK Dementia Research Institute (UK DRI Ltd; UKDRI-1014 and UKDRI-CIP0202 held by F.K.W.) through UK DRI Ltd, principally funded by the 10.13039/501100000265UK Medical Research Council. F.K.W. was also supported by an Alzheimer's Research UK Senior Research Fellowship (ARUK-SRF2018A-001). S.-H.W. received support from an Alzheimer's Research UK Senior Research Fellowship (ARUK-SRF2018B-009) and a Tenovus pilot grant (E23-19). J.K. was supported by the 10.13039/501100017510UK Dementia Research Institute, through UK DRI Ltd, principally funded by the 10.13039/501100000265Medical Research Council, by funding from the Cure Alzheimer’s Fund. K.H. is supported by a Wellcome Trust Early-Career Award (306380/Z/23/Z). The AD-SOLES project received funding from an ARUK Pilot Grant (ARUK-PPG2020A-029). L.K. is supported by the 10.13039/501100000324Gatsby Charitable Foundation and the 10.13039/100010269Wellcome Trust (via core funder of Sainsbury Wellcome Centre).

The funders had no role in study design, data collection and analysis, decision to publish, or preparation of the manuscript.

## Author contributions

S.B., L.K., J.K., S.-H.W., and F.K.W. contributed to the first draft and editing of this manuscript. K.H. contributed to the revision of this manuscript.

## Declaration of interests

F.K.W. has undertaken for fee consultancy for Alnylam Pharmaceuticals and TRIMTECH THERAPEUTICS for work unconnected to this manuscript. She also sits on the Science Advisory Board for Cambridge Phenotyping and is a member of the Executive Board of the MRC National Mouse Genetics Network and the Trisomy 21 Research Society. J.K. is CEO, founder, and shareholder of Cambridge Phenotyping.

## References

[1] Hub D.S. (2022). Statistics about dementia. Dementia Statistics Hub.

[2] Hardy J. (2025). Milestone Review: The History of Molecular Genetics Analysis of Alzheimer’s Disease. J. Neurochem..

[3] DeTure M.A., Dickson D.W. (2019). The neuropathological diagnosis of Alzheimer’s disease. Mol. Neurodegener..

[4] Masters C.L., Bateman R., Blennow K., Rowe C.C., Sperling R.A., Cummings J.L. (2015). Alzheimer’s disease. Nat. Rev. Dis. Primers.

[5] Jack C.R., Knopman D.S., Jagust W.J., Shaw L.M., Aisen P.S., Weiner M.W., Petersen R.C., Trojanowski J.Q. (2010). Hypothetical model of dynamic biomarkers of the Alzheimer’s pathological cascade. Lancet Neurol..

[6] Tromp D., Dufour A., Lithfous S., Pebayle T., Després O. (2015). Episodic memory in normal aging and Alzheimer disease: Insights from imaging and behavioral studies. Ageing Res. Rev..

[7] Bird C.M., Chan D., Hartley T., Pijnenburg Y.A., Rossor M.N., Burgess N. (2010). Topographical short-term memory differentiates Alzheimer’s disease from frontotemporal lobar degeneration. Hippocampus.

[8] Chan D., Gallaher L.M., Moodley K., Minati L., Burgess N., Hartley T. (2016). The 4 Mountains Test: A Short Test of Spatial Memory with High Sensitivity for the Diagnosis of Pre-dementia Alzheimer’s Disease. J. Vis. Exp..

[9] Newton C., Pope M., Rua C., Henson R., Ji Z., Burgess N., Rodgers C.T., Stangl M., Dounavi M., Castegnaro A. (2024). Entorhinal-based path integration selectively predicts midlife risk of Alzheimer’s disease. Alzheimer’s Dement..

[10] Guarino A., Favieri F., Boncompagni I., Agostini F., Cantone M., Casagrande M. (2019). Executive Functions in Alzheimer Disease: A Systematic Review. Front. Aging Neurosci..

[11] Ferris S.H., Farlow M. (2013). Language impairment in Alzheimer’s disease and benefits of acetylcholinesterase inhibitors. Clin. Interv. Aging.

[12] Kumar A., Sidhu J., Lui F., Tsao J.W., Abdelsattar M., Abernethy L.T., Ackley W.B., Adolphe T.S., Aeby T.C., Agasthi P., Agnihotri R., Ahmad M., Ahmad S. (2025). StatPearls.

[13] (2022). Alzheimer’s disease facts and figures (2022). Alzheimer’s & Dementia.

[14] Ismail Z., Gatchel J., Bateman D.R., Barcelos-Ferreira R., Cantillon M., Jaeger J., Donovan N.J., Mortby M.E., Mortby M.E., Ismail Z. (2018). Affective and emotional dysregulation as pre-dementia risk markers: exploring the mild behavioral impairment symptoms of depression, anxiety, irritability, and euphoria. Int. Psychogeriatr..

[15] Deardorff W.J., Grossberg G.T. (2019). Behavioral and psychological symptoms in Alzheimer’s dementia and vascular dementia. Handb. Clin. Neurol..

[16] Ismail Z., Creese B., Aarsland D., Kales H.C., Lyketsos C.G., Sweet R.A., Ballard C. (2022). Psychosis in Alzheimer disease — mechanisms, genetics and therapeutic opportunities. Nat. Rev. Neurol..

[17] Botto R., Callai N., Cermelli A., Causarano L., Rainero I. (2022). Anxiety and depression in Alzheimer’s disease: a systematic review of pathogenetic mechanisms and relation to cognitive decline. Neurol. Sci..

[18] Livingston G., Huntley J., Liu K.Y., Costafreda S.G., Selbæk G., Alladi S., Ames D., Banerjee S., Burns A., Brayne C. (2024). Dementia prevention, intervention, and care: 2024 report of the Lancet standing Commission. Lancet.

[19] Joe E., Ringman J.M. (2019). Cognitive symptoms of Alzheimer’s disease: clinical management and prevention. BMJ.

[20] Rabinovici G.D., Selkoe D.J., Schindler S.E., Aisen P., Apostolova L.G., Atri A., Greenberg S.M., Hendrix S.B., Petersen R.C., Weiner M. (2025). Donanemab: Appropriate use recommendations. J. Preven. Alzheimers Dis..

[21] Cummings J., Apostolova L., Rabinovici G.D., Atri A., Aisen P., Greenberg S., Hendrix S., Selkoe D., Weiner M., Petersen R.C. (2023). Lecanemab: Appropriate Use Recommendations. J Prev Alzheimers Dis.

[22] Games D., Adams D., Alessandrini R., Barbour R., Borthelette P., Blackwell C., Carr T., Clemens J., Donaldson T., Gillespie F. (1995). Alzheimer-type neuropathology in transgenic mice overexpressing V717F β-amyloid precursor protein. Nature.

[23] Bard F., Cannon C., Barbour R., Burke R.L., Games D., Grajeda H., Guido T., Hu K., Huang J., Johnson-Wood K. (2000). Peripherally administered antibodies against amyloid beta-peptide enter the central nervous system and reduce pathology in a mouse model of Alzheimer disease. Nat Med.

[24] DeMattos R.B., Bales K.R., Cummins D.J., Dodart J.C., Paul S.M., Holtzman D.M. (2001). Peripheral anti-A beta antibody alters CNS and plasma A beta clearance and decreases brain A beta burden in a mouse model of Alzheimer’s disease. Proc. Natl. Acad. Sci. USA.

[25] Dodart J.-C., Bales K.R., Gannon K.S., Greene S.J., DeMattos R.B., Mathis C., DeLong C.A., Wu S., Wu X., Holtzman D.M. (2002). Immunization reverses memory deficits without reducing brain Abeta burden in Alzheimer’s disease model. Nat. Neurosci..

[26] Wilcock D.M., Rojiani A., Rosenthal A., Subbarao S., Freeman M.J., Gordon M.N., Morgan D. (2004). Passive immunotherapy against Abeta in aged APP-transgenic mice reverses cognitive deficits and depletes parenchymal amyloid deposits in spite of increased vascular amyloid and microhemorrhage. J. Neuroinflammation.

[27] Racke M.M., Boone L.I., Hepburn D.L., Parsadainian M., Bryan M.T., Ness D.K., Piroozi K.S., Jordan W.H., Brown D.D., Hoffman W.P. (2005). Exacerbation of cerebral amyloid angiopathy-associated microhemorrhage in amyloid precursor protein transgenic mice by immunotherapy is dependent on antibody recognition of deposited forms of amyloid beta. J. Neurosci..

[28] Herzig M.C., Winkler D.T., Burgermeister P., Pfeifer M., Kohler E., Schmidt S.D., Danner S., Abramowski D., Stürchler-Pierrat C., Bürki K. (2004). Abeta is targeted to the vasculature in a mouse model of hereditary cerebral hemorrhage with amyloidosis. Nat. Neurosci..

[29] Yue F., Cheng Y., Breschi A., Vierstra J., Wu W., Ryba T., Sandstrom R., Ma Z., Davis C., Pope B.D. (2014). A comparative encyclopedia of DNA elements in the mouse genome. Nature.

[30] Bellenguez C., Küçükali F., Jansen I.E., Kleineidam L., Moreno-Grau S., Amin N., Naj A.C., Campos-Martin R., Grenier-Boley B., Andrade V. (2022). New insights into the genetic etiology of Alzheimer’s disease and related dementias. Nat. Genet..

[31] Reid M.M., Menon S., Liu H., Zhou H., Hu Z., Frerich S., Ding B., Oveisgharan S., Zhang Z., Nelson S. (2025). Human brain vascular multi-omics elucidates disease-risk associations. Neuron.

[32] Lee H., Pearse R.V., Lish A.M., Pan C., Augur Z.M., Terzioglu G., Gaur P., Liao M., Fujita M., Tio E.S. (2025). Contributions of Genetic Variation in Astrocytes to Cell and Molecular Mechanisms of Risk and Resilience to Late-Onset Alzheimer’s Disease. Glia.

[33] Serneels L., T’Syen D., Perez-Benito L., Theys T., Holt M.G., De Strooper B. (2020). Modeling the β-secretase cleavage site and humanizing amyloid-beta precursor protein in rat and mouse to study Alzheimer’s disease. Mol. Neurodegener..

[34] Ueno H., Yamaguchi T., Fukunaga S., Okada Y., Yano Y., Hoshino M., Matsuzaki K. (2014). Comparison between the Aggregation of Human and Rodent Amyloid β-Proteins in GM1 Ganglioside Clusters. Biochemistry.

[35] Janke C., Beck M., Stahl T., Holzer M., Brauer K., Bigl V., Arendt T. (1999). Phylogenetic diversity of the expression of the microtubule-associated protein tau: implications for neurodegenerative disorders. Brain Res Mol Brain Res.

[36] Takuma H., Arawaka S., Mori H. (2003). Isoforms changes of tau protein during development in various species. Brain Res Dev Brain Res.

[37] Jankowsky J.L., Zheng H. (2017). Practical considerations for choosing a mouse model of Alzheimer’s disease. Mol. Neurodegener..

[38] DeFranco J.P., Telling G.C. (2025). The Evolution of Experimental Rodent Models for Prion Diseases. J. Neurochem..

[39] Farshim P.P., Bates G.P. (2018). Mouse Models of Huntington’s Disease. Methods Mol. Biol..

[40] De Strooper B., Karran E. (2016). The Cellular Phase of Alzheimer’s Disease. Cell.

[41] Kanton S., Boyle M.J., He Z., Santel M., Weigert A., Sanchís-Calleja F., Guijarro P., Sidow L., Fleck J.S., Han D. (2019). Organoid single-cell genomic atlas uncovers human-specific features of brain development. Nature.

[42] Wong H.H.-W., Chou C.Y.C., Watt A.J., Sjöström P.J. (2023). Comparing mouse and human brains. eLife.

[43] Bakken T.E., Jorstad N.L., Hu Q., Lake B.B., Tian W., Kalmbach B.E., Crow M., Hodge R.D., Krienen F.M., Sorensen S.A. (2021). Comparative cellular analysis of motor cortex in human, marmoset and mouse. Nature.

[44] Loomba S., Straehle J., Gangadharan V., Heike N., Khalifa A., Motta A., Ju N., Sievers M., Gempt J., Meyer H.S. (2022). Connectomic comparison of mouse and human cortex. Science.

[45] Zhang K., Sejnowski T.J. (2000). A universal scaling law between gray matter and white matter of cerebral cortex. Proc. Natl. Acad. Sci. USA.

[46] Krienen F.M., Goldman M., Zhang Q., C H Del Rosario R., Florio M., Machold R., Saunders A., Levandowski K., Zaniewski H., Schuman B. (2020). Innovations present in the primate interneuron repertoire. Nature.

[47] Smith A.J., Clutton R.E., Lilley E., Hansen K.E.A., Brattelid T. (2018). PREPARE: guidelines for planning animal research and testing. Lab Anim.

[48] Graham M.L., Prescott M.J. (2015). The multifactorial role of the 3Rs in shifting the harm-benefit analysis in animal models of disease. Eur. J. Pharmacol..

[49] Willner P. (1990). Animal models of depression: An overview. Pharmacol. Ther..

[50] Liu H., Marsh T.W., Shi X., Renton A.E., Bowling K.M., Ziegemeier E., Wang G., Cao Y., Aristel A., Li J. (2025). The landscape of autosomal-dominant Alzheimer’s disease: global distribution and age of onset. Brain.

[51] Fernández S.G., Oria C.G., Petit D., Annaert W., Ringman J.M., Fox N.C., Ryan N.S., Chávez-Gutiérrez L. (2025). Spectrum of γ-Secretase dysfunction as a unifying predictor of ADAD age at onset across PSEN1, PSEN2 and APP causal genes. Mol. Neurodegener..

[52] Gorijala P., Aslam M.M., Dang L.-H.T., Xicota L., Fernandez M.V., Sung Y.J., Fan K.-H., Feingold E., Surace E.I., Chhatwal J.P. (2024). Alzheimer’s polygenic risk scores are associated with cognitive phenotypes in Down syndrome. Alzheimers Dement..

[53] Sawa M., Overk C., Becker A., Derse D., Albay R., Weldy K., Salehi A., Beach T.G., Doran E., Head E. (2022). Impact of increased APP gene dose in Down syndrome and the Dp16 mouse model. Alzheimers Dement..

[54] Strang K.H., Golde T.E., Giasson B.I. (2019). MAPT mutations, tauopathy, and mechanisms of neurodegeneration. Lab. Invest..

[55] Research Models | ALZFORUM. https://www.alzforum.org/research-models.

[56] Sasaguri H., Hashimoto S., Watamura N., Sato K., Takamura R., Nagata K., Tsubuki S., Ohshima T., Yoshiki A., Sato K. (2022). Recent Advances in the Modeling of Alzheimer’s Disease. Front. Neurosci..

[57] Sleegers K., Brouwers N., Gijselinck I., Theuns J., Goossens D., Wauters J., Del-Favero J., Cruts M., van Duijn C.M., Van Broeckhoven C. (2006). APP duplication is sufficient to cause early onset Alzheimer’s dementia with cerebral amyloid angiopathy. Brain.

[58] Sasmita A.O., Ong E.C., Nazarenko T., Mao S., Komarek L., Thalmann M., Hantakova V., Spieth L., Berghoff S.A., Barr H.J. (2025). Parental origin of transgene modulates amyloid-β plaque burden in the 5xFAD mouse model of Alzheimer’s disease. Neuron.

[59] Tosh J.L., Rickman M., Rhymes E., Norona F.E., Clayton E., Mucke L., Isaacs A.M., Fisher E.M.C., Wiseman F.K. (2017). The integration site of the APP transgene in the J20 mouse model of Alzheimer’s disease. Wellcome Open Res..

[60] Goodwin L.O., Splinter E., Davis T.L., Urban R., He H., Braun R.E., Chesler E.J., Kumar V., Van Min M., Ndukum J. (2019). Large-scale discovery of mouse transgenic integration sites reveals frequent structural variation and insertional mutagenesis. Genome Res..

[61] Gamache J., Benzow K., Forster C., Kemper L., Hlynialuk C., Furrow E., Ashe K.H., Koob M.D. (2019). Factors other than hTau overexpression that contribute to tauopathy-like phenotype in rTg4510 mice. Nat. Commun..

[62] Sasaguri H., Nilsson P., Hashimoto S., Nagata K., Saito T., De Strooper B., Hardy J., Vassar R., Winblad B., Saido T.C. (2017). APP mouse models for Alzheimer’s disease preclinical studies. EMBO J..

[63] Saito T., Matsuba Y., Mihira N., Takano J., Nilsson P., Itohara S., Iwata N., Saido T.C. (2014). Single App knock-in mouse models of Alzheimer’s disease. Nat. Neurosci..

[64] Saito T., Matsuba Y., Yamazaki N., Hashimoto S., Saido T.C. (2016). Calpain Activation in Alzheimer’s Model Mice Is an Artifact of APP and Presenilin Overexpression. J. Neurosci..

[65] Sasmita A.O., Depp C., Nazarenko T., Sun T., Siems S.B., Ong E.C., Nkeh Y.B., Böhler C., Yu X., Bues B. (2024). Oligodendrocytes produce amyloid-β and contribute to plaque formation alongside neurons in Alzheimer’s disease model mice. Nat. Neurosci..

[66] Sato K., Watamura N., Fujioka R., Mihira N., Sekiguchi M., Nagata K., Ohshima T., Saito T., Saido T.C., Sasaguri H. (2021). A third-generation mouse model of Alzheimer’s disease shows early and increased cored plaque pathology composed of wild-type human amyloid β peptide. J. Biol. Chem..

[67] Reaume A.G., Howland D.S., Trusko S.P., Savage M.J., Lang D.M., Greenberg B.D., Siman R., Scott R.W. (1996). Enhanced Amyloidogenic Processing of the β-Amyloid Precursor Protein in Gene-targeted Mice Bearing the Swedish Familial Alzheimer’s Disease Mutations and a “Humanized” Aβ Sequence. J. Biol. Chem..

[68] Morito T., Qi M., Kamano N., Sasaguri H., Bez S., Foiani M., Duff K., Benner S., Endo T., Hama H. (2025). Human MAPT knockin mouse models of frontotemporal dementia for the neurodegenerative research community. Cell Rep. Methods.

[69] Watamura N., Foiani M.S., Bez S., Bourdenx M., Santambrogio A., Frodsham C., Camporesi E., Brinkmalm G., Zetterberg H., Patel S. (2025). In vivo hyperphosphorylation of tau is associated with synaptic loss and behavioral abnormalities in the absence of tau seeds. Nat. Neurosci..

[70] Barman A., Schürer S., Prabhakar R. (2011). Computational modeling of substrate specificity and catalysis of the β-secretase (BACE1) enzyme. Biochemistry.

[71] Wang J., Gleeson P.A., Fourriere L. (2024). Spatial-Temporal Mapping Reveals the Golgi as the Major Processing Site for the Pathogenic Swedish APP Mutation: Familial APP Mutant Shifts the Major APP Processing Site. Traffic.

[72] Nilsberth C., Westlind-Danielsson A., Eckman C.B., Condron M.M., Axelman K., Forsell C., Stenh C., Luthman J., Teplow D.B., Younkin S.G. (2001). The “Arctic” APP mutation (E693G) causes Alzheimer’s disease by enhanced Abeta protofibril formation. Nat. Neurosci..

[73] Yang Y., Zhang W., Murzin A.G., Schweighauser M., Huang M., Lövestam S., Peak-Chew S.Y., Saito T., Saido T.C., Macdonald J. (2023). Cryo-EM structures of amyloid-β filaments with the Arctic mutation (E22G) from human and mouse brains. Acta Neuropathol..

[74] Sasner M., Preuss C., Pandey R.S., Uyar A., Garceau D., Kotredes K.P., Williams H., Oblak A.L., Lin P.B., Perkins B. (2024). In vivo validation of late-onset Alzheimer’s disease genetic risk factors. Alzheimers Dement..

[75] Koller E.J., Comstock M., Bean J.C., Escobedo G., Park K.-W., Jankowsky J.L. (2022). Temporal and spatially controlled APP transgene expression using Cre-dependent alleles. Dis. Model. Mech..

[76] Preman P., Tcw J., Calafate S., Snellinx A., Alfonso-Triguero M., Corthout N., Munck S., Thal D.R., Goate A.M., De Strooper B. (2021). Human iPSC-derived astrocytes transplanted into the mouse brain undergo morphological changes in response to amyloid-β plaques. Mol. Neurodegener..

[77] Espuny-Camacho I., Arranz A.M., Fiers M., Snellinx A., Ando K., Munck S., Bonnefont J., Lambot L., Corthout N., Omodho L. (2017). Hallmarks of Alzheimer’s Disease in Stem-Cell-Derived Human Neurons Transplanted into Mouse Brain. Neuron.

[78] Mancuso R., Van Den Daele J., Fattorelli N., Wolfs L., Balusu S., Burton O., Liston A., Sierksma A., Fourne Y., Poovathingal S. (2019). Stem-cell-derived human microglia transplanted in mouse brain to study human disease. Nat. Neurosci..

[79] Hasselmann J., Coburn M.A., England W., Figueroa Velez D.X., Kiani Shabestari S., Tu C.H., McQuade A., Kolahdouzan M., Echeverria K., Claes C. (2019). Development of a Chimeric Model to Study and Manipulate Human Microglia In Vivo. Neuron.

[80] Rathinam C., Poueymirou W.T., Rojas J., Murphy A.J., Valenzuela D.M., Yancopoulos G.D., Rongvaux A., Eynon E.E., Manz M.G., Flavell R.A. (2011). Efficient differentiation and function of human macrophages in humanized CSF-1 mice. Blood.

[81] Jucker M., Walker L.C. (2013). Self-propagation of pathogenic protein aggregates in neurodegenerative diseases. Nature.

[82] Gibbs M.E., Maksel D., Gibbs Z., Hou X., Summers R.J., Small D.H. (2010). Memory loss caused by beta-amyloid protein is rescued by a beta(3)-adrenoceptor agonist. Neurobiol. Aging.

[83] Tucci P., Mhillaj E., Morgese M.G., Colaianna M., Zotti M., Schiavone S., Cicerale M., Trezza V., Campolongo P., Cuomo V. (2014). Memantine prevents memory consolidation failure induced by soluble beta amyloid in rats. Front. Behav. Neurosci..

[84] Rossi Daré L., Garcia A., Neves B.-H., Mello-Carpes P.B. (2020). One physical exercise session promotes recognition learning in rats with cognitive deficits related to amyloid beta neurotoxicity. Brain Res..

[85] Finnie P.S.B., Nader K. (2020). Amyloid Beta Secreted during Consolidation Prevents Memory Malleability. Curr. Biol..

[86] Jin M., Xu R., Wang L., Alam M.M., Ma Z., Zhu S., Martini A.C., Jadali A., Bernabucci M., Xie P. (2022). Type-I-interferon signaling drives microglial dysfunction and senescence in human iPSC models of Down syndrome and Alzheimer’s disease. Cell Stem Cell.

[87] Åhlgren J., Voikar V. (2019). Experiments done in Black-6 mice: what does it mean?. Lab Anim.

[88] Specht C.G., Schoepfer R. (2001). Deletion of the alpha-synuclein locus in a subpopulation of C57BL/6J inbred mice. BMC Neurosci..

[89] Chang B., Hurd R., Wang J., Nishina P. (2013). Survey of common eye diseases in laboratory mouse strains. Investig. Ophthalmol. Vis. Sci..

[90] Mattapallil M.J., Wawrousek E.F., Chan C.-C., Zhao H., Roychoudhury J., Ferguson T.A., Caspi R.R. (2012). The Rd8 mutation of the Crb1 gene is present in vendor lines of C57BL/6N mice and embryonic stem cells, and confounds ocular induced mutant phenotypes. Investig. Ophthalmol. Vis. Sci..

[91] Retinal degeneration 1 pde6brd1. https://www.jax.org/research-and-faculty/resources/eye-mutant-resource/retinal-degeneration-1-pde6brd1.

[92] Ippati S., Matthias Ittner L., Diana Ke Y., Ferretti M.T., Dimech A.S., Chadha A.S. (2021). Sex and Gender Differences in Alzheimer’s Disease.

[93] Wang J., Tanila H., Puoliväli J., Kadish I., van Groen T. (2003). Gender differences in the amount and deposition of amyloidbeta in APPswe and PS1 double transgenic mice. Neurobiol. Dis..

[94] Jiao S.-S., Bu X.-L., Liu Y.-H., Zhu C., Wang Q.-H., Shen L.-L., Liu C.-H., Wang Y.-R., Yao X.-Q., Wang Y.-J. (2016). Sex Dimorphism Profile of Alzheimer’s Disease-Type Pathologies in an APP/PS1 Mouse Model. Neurotox. Res..

[95] Sil A., Erfani A., Lamb N., Copland R., Riedel G., Platt B. (2022). Sex Differences in Behavior and Molecular Pathology in the 5XFAD Model. J. Alzheimers Dis..

[96] O’Leary T.P., Mantolino H.M., Stover K.R., Brown R.E. (2020). Age-related deterioration of motor function in male and female 5xFAD mice from 3 to 16 months of age. Gene Brain Behav..

[97] Lansdell T.A., Xu H., Galligan J.J., Dorrance A.M. (2023). Effects of Striatal Amyloidosis on the Dopaminergic System and Behavior: A Comparative Study in Male and Female 5XFAD Mice. J. Alzheimers Dis..

[98] Pervolaraki E., Hall S.P., Foresteire D., Saito T., Saido T.C., Whittington M.A., Lever C., Dachtler J. (2019). Insoluble Aβ overexpression in an App knock-in mouse model alters microstructure and gamma oscillations in the prefrontal cortex, affecting anxiety-related behaviours. Dis. Model. Mech..

[99] Masuda A., Kobayashi Y., Kogo N., Saito T., Saido T.C., Itohara S. (2016). Cognitive deficits in single App knock-in mouse models. Neurobiol. Learn. Mem..

[100] Biechele G., Franzmeier N., Blume T., Ewers M., Luque J.M., Eckenweber F., Sacher C., Beyer L., Ruch-Rubinstein F., Lindner S. (2020). Glial activation is moderated by sex in response to amyloidosis but not to tau pathology in mouse models of neurodegenerative diseases. J. Neuroinflammation.

[101] Hair K., Wilson E., Maksym O., Macleod M.R., Sena E.S. (2024). A Systematic Online Living Evidence Summary of experimental Alzheimer’s disease research. J. Neurosci. Methods.

[102] Kraeuter A.-K., Guest P.C., Sarnyai Z. (2019). The Y-Maze for Assessment of Spatial Working and Reference Memory in Mice. Methods Mol. Biol..

[103] Deacon R.M.J., Rawlins J.N.P. (2006). T-maze alternation in the rodent. Nat. Protoc..

[104] Barnes C.A. (1988). Aging and the physiology of spatial memory. Neurobiol. Aging.

[105] Morris R. (1984). Developments of a water-maze procedure for studying spatial learning in the rat. J. Neurosci. Methods.

[106] Iivonen H., Nurminen L., Harri M., Tanila H., Puoliväli J. (2003). Hypothermia in mice tested in Morris water maze. Behav. Brain Res..

[107] Harrison F.E., Hosseini A.H., McDonald M.P. (2009). Endogenous anxiety and stress responses in water maze and Barnes maze spatial memory tasks. Behav. Brain Res..

[108] Youngstrom I.A., Strowbridge B.W. (2012). Visual landmarks facilitate rodent spatial navigation in virtual reality environments. Learn. Mem..

[109] Thurley K., Ayaz A. (2017). Virtual reality systems for rodents. Curr. Zool..

[110] Vogel-Ciernia A., Wood M.A. (2014). Examining object location and object recognition memory in mice. Curr. Protoc. Neurosci..

[111] Crawley J.N. (2004). Designing mouse behavioral tasks relevant to autistic-like behaviors. Ment. Retard. Dev. Disabil. Res. Rev..

[112] Filali M., Lalonde R., Rivest S. (2011). Anomalies in social behaviors and exploratory activities in an APPswe/PS1 mouse model of Alzheimer’s disease. Physiol. Behav..

[113] Huston J.P., Chao O.Y. (2023). Probing the nature of episodic memory in rodents. Neurosci. Biobehav. Rev..

[114] Horner A.E., Heath C.J., Hvoslef-Eide M., Kent B.A., Kim C.H., Nilsson S.R.O., Alsiö J., Oomen C.A., Holmes A., Saksida L.M. (2013). The touchscreen operant platform for testing learning and memory in rats and mice. Nat. Protoc..

[115] Barnett J.H., Blackwell A.D., Sahakian B.J., Robbins T.W. (2016). The Paired Associates Learning (PAL) Test: 30 Years of CANTAB Translational Neuroscience from Laboratory to Bedside in Dementia Research. Curr. Top Behav. Neurosci..

[116] Pettigrew C., Soldan A., Brichko R., Zhu Y., Wang M.-C., Kutten K., Bilgel M., Mori S., Miller M.I., Albert M. (2022). Computerized paired associate learning performance and imaging biomarkers in older adults without dementia. Brain Imaging Behav..

[117] Baker J.E., Pietrzak R.H., Laws S.M., Ames D., Villemagne V.L., Rowe C.C., Masters C.L., Maruff P., Lim Y.Y. (2019). Visual paired associate learning deficits associated with elevated beta-amyloid in cognitively normal older adults. Neuropsychology.

[118] Tozzi F., Guglielmo S., Paraciani C., van den Oever M.C., Mainardi M., Cattaneo A., Origlia N. (2024). Involvement of a lateral entorhinal cortex engram in episodic-like memory recall. Cell Rep..

[119] Broadbelt T., Mutlu-Smith M., Carnicero-Senabre D., Saido T.C., Saito T., Wang S.-H. (2022). Impairment in novelty-promoted memory via behavioral tagging and capture before apparent memory loss in a knock-in model of Alzheimer’s disease. Sci. Rep..

[120] Idowu M.I., Szameitat A.J., Parton A. (2024). The assessment of executive function abilities in healthy and neurodegenerative aging-A selective literature review. Front. Aging Neurosci..

[121] Romberg C., Horner A.E., Bussey T.J., Saksida L.M. (2013). A touch screen-automated cognitive test battery reveals impaired attention, memory abnormalities, and increased response inhibition in the TgCRND8 mouse model of Alzheimer’s disease. Neurobiol. Aging.

[122] Romberg C., Mattson M.P., Mughal M.R., Bussey T.J., Saksida L.M. (2011). Impaired attention in the 3xTgAD mouse model of Alzheimer’s disease: rescue by donepezil (Aricept). J. Neurosci..

[123] Mar A.C., Horner A.E., Nilsson S.R.O., Alsiö J., Kent B.A., Kim C.H., Holmes A., Saksida L.M., Bussey T.J. (2013). The touchscreen operant platform for assessing executive function in rats and mice. Nat. Protoc..

[124] Beraldo F.H., Palmer D., Memar S., Wasserman D.I., Lee W.-J.V., Liang S., Creighton S.D., Kolisnyk B., Cowan M.F., Mels J. (2019). MouseBytes, an open-access high-throughput pipeline and database for rodent touchscreen-based cognitive assessment. eLife.

[125] Dumont J.R., Sheppard P.A.S., Fodor C., Coto M.A., Yang S., Saito T., Saido T.C., Rylett R.J., Prado M.A.M., Bussey T.J. (2025). Impaired Cognitive Flexibility With Preserved Learning in an Amyloid Precursor Protein Knock-In Mouse Model of Amyloidopathy. Gene Brain Behav..

[126] Saifullah M.A.B., Komine O., Dong Y., Fukumoto K., Sobue A., Endo F., Saito T., Saido T.C., Yamanaka K., Mizoguchi H. (2020). Touchscreen-based location discrimination and paired associate learning tasks detect cognitive impairment at an early stage in an App knock-in mouse model of Alzheimer’s disease. Mol. Brain.

[127] Shepherd A., Lim J.K.H., Wong V.H.Y., Zeleznikow-Johnston A.M., Churilov L., Nguyen C.T.O., Bui B.V., Hannan A.J., Burrows E.L. (2021). Progressive impairments in executive function in the APP/PS1 model of Alzheimer’s disease as measured by translatable touchscreen testing. Neurobiol. Aging.

[128] Van den Broeck L., Hansquine P., Callaerts-Vegh Z., D’Hooge R. (2019). Impaired Reversal Learning in APPPS1-21 Mice in the Touchscreen Visual Discrimination Task. Front. Behav. Neurosci..

[129] Urai A.E., Aguillon-Rodriguez V., Laranjeira I.C., Cazettes F., The I.B.L., Mainen Z.F., Churchland A.K. (2021). Citric Acid Water as an Alternative to Water Restriction for High-Yield Mouse Behavior. eNeuro.

[130] Reinagel P. (2024). Water restriction still has a place. Lab Anim..

[131] Kim B., Noh G.O., Kim K. (2021). Behavioural and psychological symptoms of dementia in patients with Alzheimer’s disease and family caregiver burden: a path analysis. BMC Geriatr..

[132] Zhang N.K., Zhang S.K., Zhang L.I., Tao H.W., Zhang G.-W. (2024). The neural basis of neuropsychiatric symptoms in Alzheimer’s disease. Front. Aging Neurosci..

[133] La-Vu M., Tobias B.C., Schuette P.J., Adhikari A. (2020). To Approach or Avoid: An Introductory Overview of the Study of Anxiety Using Rodent Assays. Front. Behav. Neurosci..

[134] Johnson A.R., Christensen B.A., Kelly S.J., Calipari E.S. (2022). The influence of reinforcement schedule on experience-dependent changes in motivation. J. Exp. Anal. Behav..

[135] Hamaguchi T., Tsutsui-Kimura I., Mimura M., Saito T., Saido T.C., Tanaka K.F. (2019). AppNL-G-F/NL-G-F mice overall do not show impaired motivation, but cored amyloid plaques in the striatum are inversely correlated with motivation. Neurochem. Int..

[136] Vaquero-Puyuelo D., De-la-Cámara C., Olaya B., Gracia-García P., Lobo A., López-Antón R., Santabárbara J. (2021). Anhedonia as a Potential Risk Factor of Alzheimer’s Disease in a Community-Dwelling Elderly Sample: Results from the ZARADEMP Project. Int. J. Environ. Res. Publ. Health.

[137] Saz P., López-Antón R., Dewey M.E., Ventura T., Martín A., Marcos G., De La Cámara C., Quintanilla M.A., Quetglas B., Bel M. (2009). Prevalence and implications of psychopathological non-cognitive symptoms in dementia. Acta Psychiatr. Scand..

[138] Liu M.-Y., Yin C.-Y., Zhu L.-J., Zhu X.-H., Xu C., Luo C.-X., Chen H., Zhu D.-Y., Zhou Q.-G. (2018). Sucrose preference test for measurement of stress-induced anhedonia in mice. Nat. Protoc..

[139] Gilley D.W., Wilson R.S., Beckett L.A., Evans D.A. (1997). Psychotic Symptoms and Physically Aggressive Behavior in Alzheimer’s Disease. J. Am. Geriatr. Soc..

[140] Hu Y., Niu L., Chen Y., Yang H., Qiu X., Jiang F., Liu C., Cai H., Le W. (2025). Voluntary wheel running exercise improves sleep disorder, circadian rhythm disturbance, and neuropathology in an animal model of Alzheimer’s disease. Alzheimer's Dement..

[141] Yang H., Niu L., Tian L., Hu Y., Cheng C., Li S., Le W. (2025). Circadian rhythm disturbances in Alzheimer’s disease: insights from plaque-free and plaque-burdened stages in APPSWE/PS1dE9 mice. Alzheimers Res. Ther..

[142] Britz J., Ojo E., Dhukhwa A., Saito T., Saido T.C., Hascup E.R., Hascup K.N., Tischkau S.A. (2022). Assessing Sex-Specific Circadian, Metabolic, and Cognitive Phenotypes in the AβPP/PS1 and APPNL-F/NL-F Models of Alzheimer’s Disease. J. Alzheimers Dis..

[143] Vloeberghs E., Van Dam D., Engelborghs S., Nagels G., Staufenbiel M., De Deyn P.P. (2004). Altered circadian locomotor activity in APP23 mice: a model for BPSD disturbances. Eur. J. Neurosci..

[144] Ognibene E., Middei S., Daniele S., Adriani W., Ghirardi O., Caprioli A., Laviola G. (2005). Aspects of spatial memory and behavioral disinhibition in Tg2576 transgenic mice as a model of Alzheimer’s disease. Behav. Brain Res..

[145] Ambrée O., Touma C., Görtz N., Keyvani K., Paulus W., Palme R., Sachser N. (2006). Activity changes and marked stereotypic behavior precede Abeta pathology in TgCRND8 Alzheimer mice. Neurobiol. Aging.

[146] Position paper: Forced swim test (September 2024) | NC3Rs. https://nc3rs.org.uk/position-paper-forced-swim-test-september-2024.

[147] Deacon R.M.J. (2006). Assessing nest building in mice. Nat. Protoc..

[148] Deacon R.M.J. (2006). Digging and marble burying in mice: simple methods for in vivo identification of biological impacts. Nat. Protoc..

[149] Keszycki R., Rodriguez G., Dunn J.T., Locci A., Orellana H., Haupfear I., Dominguez S., Fisher D.W., Dong H. (2023). Characterization of apathy-like behaviors in the 5xFAD mouse model of Alzheimer’s disease. Neurobiol. Aging.

[150] Santana-Santana M., Bayascas J.-R., Giménez-Llort L. (2021). Sex-Dependent Signatures, Time Frames and Longitudinal Fine-Tuning of the Marble Burying Test in Normal and AD-Pathological Aging Mice. Biomedicines.

[151] Deacon R.M.J., Cholerton L.L., Talbot K., Nair-Roberts R.G., Sanderson D.J., Romberg C., Koros E., Bornemann K.D., Rawlins J.N.P. (2008). Age-dependent and -independent behavioral deficits in Tg2576 mice. Behav. Brain Res..

[152] Janus C., Flores A.Y., Xu G., Borchelt D.R. (2015). Behavioral abnormalities in APPSwe/PS1dE9 mouse model of AD-like pathology: comparative analysis across multiple behavioral domains. Neurobiol. Aging.

[153] Voikar V., Gaburro S. (2020). Three Pillars of Automated Home-Cage Phenotyping of Mice: Novel Findings, Refinement, and Reproducibility Based on Literature and Experience. Front. Behav. Neurosci..

[154] Bains R.S., Cater H.L., Sillito R.R., Chartsias A., Sneddon D., Concas D., Keskivali-Bond P., Lukins T.C., Wells S., Acevedo Arozena A. (2016). Analysis of Individual Mouse Activity in Group Housed Animals of Different Inbred Strains using a Novel Automated Home Cage Analysis System. Front. Behav. Neurosci..

[155] Bains R.S., Forrest H., Sillito R.R., Armstrong J.D., Stewart M., Nolan P.M., Wells S.E. (2023). Longitudinal home-cage automated assessment of climbing behavior shows sexual dimorphism and aging-related decrease in C57BL/6J healthy mice and allows early detection of motor impairment in the N171-82Q mouse model of Huntington’s disease. Front. Behav. Neurosci..

[156] Ho H., Kejzar N., Sasaguri H., Saito T., Saido T.C., De Strooper B., Bauza M., Krupic J. (2023). A fully automated home cage for long-term continuous phenotyping of mouse cognition and behavior. Cell Rep. Methods.

[157] Krackow S., Vannoni E., Codita A., Mohammed A.H., Cirulli F., Branchi I., Alleva E., Reichelt A., Willuweit A., Voikar V. (2010). Consistent behavioral phenotype differences between inbred mouse strains in the IntelliCage. Gene Brain Behav..

[158] Ma X., Schildknecht B., Steiner A.C., Amrein I., Nigri M., Bramati G., Wolfer D.P. (2023). Refinement of IntelliCage protocols for complex cognitive tasks through replacement of drinking restrictions by incentive-disincentive paradigms. Front. Behav. Neurosci..

[159] Musiek E.S., Xiong D.D., Holtzman D.M. (2015). Sleep, circadian rhythms, and the pathogenesis of Alzheimer Disease. Exp. Mol. Med..

[160] Judd J.M., Winslow W., McDonough I., Mistry F., Velazquez R. (2024). Modifying reaction time tasks parameters in the automated IntelliCage identifies heightened impulsivity and impaired attention in the 3xTg-AD model of Alzheimer’s disease. Front. Aging Neurosci..

[161] Masuda A., Kobayashi Y., Itohara S. (2018). Automated, Long-term Behavioral Assay for Cognitive Functions in Multiple Genetic Models of Alzheimer’s Disease, Using IntelliCage. J. Vis. Exp..

[162] Codita A., Gumucio A., Lannfelt L., Gellerfors P., Winblad B., Mohammed A.H., Nilsson L.N.G. (2010). Impaired behavior of female tg-ArcSwe APP mice in the IntelliCage: A longitudinal study. Behav. Brain Res..

[163] Goulding E.H., Schenk A.K., Juneja P., MacKay A.W., Wade J.M., Tecott L.H. (2008). A robust automated system elucidates mouse home cage behavioral structure. Proc. Natl. Acad. Sci. USA.

[164] Mathis A., Mamidanna P., Cury K.M., Abe T., Murthy V.N., Mathis M.W., Bethge M. (2018). DeepLabCut: markerless pose estimation of user-defined body parts with deep learning. Nat. Neurosci..

[165] Pereira T.D., Tabris N., Matsliah A., Turner D.M., Li J., Ravindranath S., Papadoyannis E.S., Normand E., Deutsch D.S., Wang Z.Y. (2022). SLEAP: A deep learning system for multi-animal pose tracking. Nat. Methods.

[166] Goodwin N.L., Choong J.J., Hwang S., Pitts K., Bloom L., Islam A., Zhang Y.Y., Szelenyi E.R., Tong X., Newman E.L. (2024). Simple Behavioral Analysis (SimBA) as a platform for explainable machine learning in behavioral neuroscience. Nat. Neurosci..

[167] Goodwin N.L., Golden S.A. (2024). Keeping it simple – a Simple Behavioral Analysis (SimBA) primer. NPP—Digit Psychiatry Neurosci.

[168] Luxem K., Mocellin P., Fuhrmann F., Kürsch J., Miller S.R., Palop J.J., Remy S., Bauer P. (2022). Identifying behavioral structure from deep variational embeddings of animal motion. Commun. Biol..

[169] Datta S.R. (2019). Q&A: Understanding the composition of behavior. BMC Biol..

[170] Lin S., Gillis W.F., Weinreb C., Zeine A., Jones S.C., Robinson E.M., Markowitz J., Datta S.R. (2024). Characterizing the structure of mouse behavior using Motion Sequencing. Nat. Protoc..

[171] Miller S.R., Luxem K., Lauderdale K., Nambiar P., Honma P.S., Ly K.K., Bangera S., Bullock M., Shin J., Kaliss N. (2024). Machine learning reveals prominent spontaneous behavioral changes and treatment efficacy in humanized and transgenic Alzheimer’s disease models. Cell Rep..

[172] Vandenabeele M., Veys L., Lemmens S., Hadoux X., Gelders G., Masin L., Serneels L., Theunis J., Saito T., Saido T.C. (2021). The AppNL-G-F mouse retina is a site for preclinical Alzheimer’s disease diagnosis and research. Acta Neuropathol. Commun..

[173] Bevan R.J., Hughes T.R., Williams P.A., Good M.A., Morgan B.P., Morgan J.E. (2020). Retinal ganglion cell degeneration correlates with hippocampal spine loss in experimental Alzheimer’s disease. Acta Neuropathol. Commun..

[174] Lalonde R., Fukuchi K.-I., Strazielle C. (2012). Neurologic and motor dysfunctions in APP transgenic mice. Rev. Neurosci..

[175] Rodgers S.P., Born H.A., Das P., Jankowsky J.L. (2012). Transgenic APP expression during postnatal development causes persistent locomotor hyperactivity in the adult. Mol. Neurodegener..

[176] Brady E.S., Griffiths J., Andrianova L., Bielska M.H., Saito T., Saido T.C., Randall A.D., Tamagnini F., Witton J., Craig M.T. (2023). Alterations to parvalbumin-expressing interneuron function and associated network oscillations in the hippocampal – medial prefrontal cortex circuit during natural sleep in AppNL-G-F/NL-G-F mice. Neurobiol. Dis..

[177] Cacucci F., Yi M., Wills T.J., Chapman P., O’Keefe J. (2008). Place cell firing correlates with memory deficits and amyloid plaque burden in Tg2576 Alzheimer mouse model. Proc. Natl. Acad. Sci..

[178] Cayzac S., Mons N., Ginguay A., Allinquant B., Jeantet Y., Cho Y.H. (2015). Altered hippocampal information coding and network synchrony in APP-PS1 mice. Neurobiol. Aging.

[179] Ciupek S.M., Cheng J., Ali Y.O., Lu H.-C., Ji D. (2015). Progressive Functional Impairments of Hippocampal Neurons in a Tauopathy Mouse Model. J. Neurosci..

[180] Gillespie A.K., Jones E.A., Lin Y.-H., Karlsson M.P., Kay K., Yoon S.Y., Tong L.M., Nova P., Carr J.S., Frank L.M. (2016). Apolipoprotein E4 Causes Age-Dependent Disruption of Slow Gamma Oscillations during Hippocampal Sharp-Wave Ripples. Neuron.

[181] Iaccarino H.F., Singer A.C., Martorell A.J., Rudenko A., Gao F., Gillingham T.Z., Mathys H., Seo J., Kritskiy O., Abdurrob F. (2016). Gamma frequency entrainment attenuates amyloid load and modifies microglia. Nature.

[182] Ittner A.A., Gladbach A., Bertz J., Suh L.S., Ittner L.M. (2014). p38 MAP kinase-mediated NMDA receptor-dependent suppression of hippocampal hypersynchronicity in a mouse model of Alzheimer’s disease. Acta Neuropathol. Commun..

[183] Jun H., Bramian A., Soma S., Saito T., Saido T.C., Igarashi K.M. (2020). Disrupted Place Cell Remapping and Impaired Grid Cells in a Knockin Model of Alzheimer’s Disease. Neuron.

[184] Mably A.J., Gereke B.J., Jones D.T., Colgin L.L. (2017). Impairments in spatial representations and rhythmic coordination of place cells in the 3xTg mouse model of Alzheimer’s disease. Hippocampus.

[185] Rubio S.E., Vega-Flores G., Martínez A., Bosch C., Pérez-Mediavilla A., Río J. del, Gruart A., Delgado-García J.M., Soriano E., Pascual M. (2012). Accelerated aging of the GABAergic septohippocampal pathway and decreased hippocampal rhythms in a mouse model of Alzheimer’s disease. FASEB J..

[186] Schneider F., Baldauf K., Wetzel W., Reymann K.G. (2014). Behavioral and EEG changes in male 5xFAD mice. Physiol. Behav..

[187] Villette V., Poindessous-Jazat F., Simon A., Léna C., Roullot E., Bellessort B., Epelbaum J., Dutar P., Stéphan A. (2010). Decreased rhythmic GABAergic septal activity and memory-associated theta oscillations after hippocampal amyloid-beta pathology in the rat. J. Neurosci..

[188] Witton J., Staniaszek L.E., Bartsch U., Randall A.D., Jones M.W., Brown J.T. (2016). Disrupted hippocampal sharp-wave ripple-associated spike dynamics in a transgenic mouse model of dementia. J. Physiol..

[189] Ying J., Keinath A.T., Lavoie R., Vigneault E., El Mestikawy S., Brandon M.P. (2022). Disruption of the grid cell network in a mouse model of early Alzheimer’s disease. Nat. Commun..

[190] Fu H., Rodriguez G.A., Herman M., Emrani S., Nahmani E., Barrett G., Figueroa H.Y., Goldberg E., Hussaini S.A., Duff K.E. (2017). Tau Pathology Induces Excitatory Neuron Loss, Grid Cell Dysfunction, and Spatial Memory Deficits Reminiscent of Early Alzheimer’s Disease. Neuron.

[191] Booth C.A., Witton J., Nowacki J., Tsaneva-Atanasova K., Jones M.W., Randall A.D., Brown J.T. (2016). Altered Intrinsic Pyramidal Neuron Properties and Pathway-Specific Synaptic Dysfunction Underlie Aberrant Hippocampal Network Function in a Mouse Model of Tauopathy. J. Neurosci..

[192] Van Erum J., Van Dam D., Sheorajpanday R., De Deyn P.P. (2019). Sleep architecture changes in the APP23 mouse model manifest at onset of cognitive deficits. Behav. Brain Res..

[193] Verret L., Mann E.O., Hang G.B., Barth A.M.I., Cobos I., Ho K., Devidze N., Masliah E., Kreitzer A.C., Mody I. (2012). Inhibitory Interneuron Deficit Links Altered Network Activity and Cognitive Dysfunction in Alzheimer Model. Cell.

[194] Zhao R., Fowler S.W., Chiang A.C.A., Ji D., Jankowsky J.L. (2014). Impairments in experience-dependent scaling and stability of hippocampal place fields limit spatial learning in a mouse model of Alzheimer’s disease. Hippocampus.

[195] Murdock M.H., Yang C.-Y., Sun N., Pao P.-C., Blanco-Duque C., Kahn M.C., Kim T., Lavoie N.S., Victor M.B., Islam M.R. (2024). Multisensory gamma stimulation promotes glymphatic clearance of amyloid. Nature.

[196] O’Keefe J., Nadel L. (1978).

[197] Buzsáki G., Moser E.I. (2013). Memory, navigation and theta rhythm in the hippocampal-entorhinal system. Nat. Neurosci..

[198] Busche M.A., Chen X., Henning H.A., Reichwald J., Staufenbiel M., Sakmann B., Konnerth A. (2012). Critical role of soluble amyloid-β for early hippocampal hyperactivity in a mouse model of Alzheimer’s disease. Proc. Natl. Acad. Sci..

[199] Busche M.A., Eichhoff G., Adelsberger H., Abramowski D., Wiederhold K.-H., Haass C., Staufenbiel M., Konnerth A., Garaschuk O. (2008). Clusters of Hyperactive Neurons Near Amyloid Plaques in a Mouse Model of Alzheimer’s Disease. Science.

[200] Wu J.W., Hussaini S.A., Bastille I.M., Rodriguez G.A., Mrejeru A., Rilett K., Sanders D.W., Cook C., Fu H., Boonen R.A.C.M. (2016). Neuronal activity enhances tau propagation and tau pathology in vivo. Nat. Neurosci..

[201] Harris S.S., Rajani R.M., Zünkler J., Ellingford R., Yang M., Rowland J.M., Schmidt A., Lee B.I., Kehring M., Hellmuth M. (2025). The amyloid precursor family of proteins in excitatory neurons are essential for regulating cortico-hippocampal circuit dynamics in vivo. Cell Rep..

[202] Harris S.S., Ellingford R., Hartmann J., Dasgupta D., Kehring M., Rajani R.M., Graykowski D., Quittot N., Sivasankaran D., Commins C. (2025). Alzheimer’s disease patient-derived high-molecular-weight tau impairs bursting in hippocampal neurons. Cell.

[203] Cheng J., Ji D. (2013). Rigid firing sequences undermine spatial memory codes in a neurodegenerative mouse model. eLife.

[204] Long X., Tao Y., Chen X.-C., Deng B., Cai J., Zhang S.-J. (2021). Getting Lost: Place Cells and Grid Cells in Rodent Models of Alzheimer’s Disease. Neurosci. Bull..

[205] Chung H., Park K., Jang H.J., Kohl M.M., Kwag J. (2020). Dissociation of somatostatin and parvalbumin interneurons circuit dysfunctions underlying hippocampal theta and gamma oscillations impaired by amyloid β oligomers in vivo. Brain Struct. Funct..

[206] Hoffman C., Cheng J., Morales R., Ji D., Dabaghian Y. (2025). Altered patterning of neural activity in a neuropathology. Sci. Rep..

[207] Hafting T., Fyhn M., Molden S., Moser M.-B., Moser E.I. (2005). Microstructure of a spatial map in the entorhinal cortex. Nature.

[208] Gil M., Ancau M., Schlesiger M.I., Neitz A., Allen K., De Marco R.J., Monyer H. (2018). Impaired path integration in mice with disrupted grid cell firing. Nat. Neurosci..

[209] Clark H., Nolan M.F. (2024). Task-anchored grid cell firing is selectively associated with successful path integration-dependent behaviour. eLife.

[210] (2018). Recommendations for the Design and Analysis of *In Vivo* Electrophysiology Studies. J. Neurosci..

[211] Steinmetz N.A., Aydin C., Lebedeva A., Okun M., Pachitariu M., Bauza M., Beau M., Bhagat J., Böhm C., Broux M. (2021). Neuropixels 2.0: A miniaturized high-density probe for stable, long-term brain recordings. Science.

[212] Pachitariu M., Sridhar S., Pennington J., Stringer C. (2024). Spike sorting with Kilosort4. Nat. Methods.

[213] Wang S., Spink A., Riedel G., Truong K., Robinson L. (2024). Proceedings the 13th International Conference on Methods and Techniques in Behavioral Research, Aberdeen, May 15-17.

[214] Dijkhuizen S., Van Ginneken L.M.C., IJpelaar A.H.C., Koekkoek S.K.E., De Zeeuw C.I., Boele H.J. (2024). Impact of enriched environment on motor performance and learning in mice. Sci. Rep..

[215] Bailoo J.D., Murphy E., Boada-Saña M., Varholick J.A., Hintze S., Baussière C., Hahn K.C., Göpfert C., Palme R., Voelkl B. (2018). Effects of Cage Enrichment on Behavior, Welfare and Outcome Variability in Female Mice. Front. Behav. Neurosci..

[216] Castro de Jesus L., S Rodrigues A.L. (2025). Non-aversive handling in laboratory animals and its effects on depressive-like and anxiety-related behaviors: A scoping review. Physiol. Behav..

[217] Wolfer D.P., Litvin O., Morf S., Nitsch R.M., Lipp H.-P., Würbel H. (2004). Laboratory animal welfare: cage enrichment and mouse behaviour. Nature.

[218] Dong H., Goico B., Martin M., Csernansky C.A., Bertchume A., Csernansky J.G. (2004). Modulation of hippocampal cell proliferation, memory, and amyloid plaque deposition in APPsw (Tg2576) mutant mice by isolation stress. Neuroscience.

[219] Pedrini S., Thomas C., Brautigam H., Schmeidler J., Ho L., Fraser P., Westaway D., Hyslop P.S., Martins R.N., Buxbaum J.D. (2009). Dietary composition modulates brain mass and solubilizable Aβ levels in a mouse model of aggressive Alzheimer’s amyloid pathology. Mol. Neurodegener..

[220] Hooijmans C.R., Rutters F., Dederen P.J., Gambarota G., Veltien A., Van Groen T., Broersen L.M., Lütjohann D., Heerschap A., Tanila H. (2007). Changes in cerebral blood volume and amyloid pathology in aged Alzheimer APP/PS1 mice on a docosahexaenoic acid (DHA) diet or cholesterol enriched Typical Western Diet (TWD). Neurobiol. Dis..

[221] Julien C., Tremblay C., Phivilay A., Berthiaume L., Emond V., Julien P., Calon F. (2010). High-fat diet aggravates amyloid-beta and tau pathologies in the 3xTg-AD mouse model. Neurobiol. Aging.

[222] Patel N.V., Gordon M.N., Connor K.E., Good R.A., Engelman R.W., Mason J., Morgan D.G., Morgan T.E., Finch C.E. (2005). Caloric restriction attenuates Abeta-deposition in Alzheimer transgenic models. Neurobiol. Aging.

[223] Halagappa V.K.M., Guo Z., Pearson M., Matsuoka Y., Cutler R.G., Laferla F.M., Mattson M.P. (2007). Intermittent fasting and caloric restriction ameliorate age-related behavioral deficits in the triple-transgenic mouse model of Alzheimer’s disease. Neurobiol. Dis..

[224] Shepherd A., Zhang T., Hoffmann L.B., Zeleznikow-Johnston A.M., Churilov L., Hannan A.J., Burrows E.L. (2021). A Preclinical Model of Computerized Cognitive Training: Touchscreen Cognitive Testing Enhances Cognition and Hippocampal Cellular Plasticity in Wildtype and Alzheimer’s Disease Mice. Front. Behav. Neurosci..

[225] Bimonte S., Barbieri A., Amruthraj N.J., Cascella M., Cuomo A., Arra C., Cascella M. (2020). General Anesthesia Research Neuromethods.

[226] Palop J.J., Chin J., Roberson E.D., Wang J., Thwin M.T., Bien-Ly N., Yoo J., Ho K.O., Yu G.-Q., Kreitzer A. (2007). Aberrant excitatory neuronal activity and compensatory remodeling of inhibitory hippocampal circuits in mouse models of Alzheimer’s disease. Neuron.

[227] Minkeviciene R., Rheims S., Dobszay M.B., Zilberter M., Hartikainen J., Fülöp L., Penke B., Zilberter Y., Harkany T., Pitkänen A. (2009). Amyloid beta-induced neuronal hyperexcitability triggers progressive epilepsy. J. Neurosci..

[228] Wang And L.A., Goonewardene Z. (2004). The use of MIXED models in the analysis of animal experiments with repeated measures data. Can. J. Anim. Sci..

[229] Walley R., Sherington J., Rastrick J., Detrait E., Hanon E., Watt G. (2016). Using Bayesian analysis in repeated preclinical in vivo studies for a more effective use of animals. Pharm. Stat..

[230] AD Knowledge Portal. https://adknowledgeportal.synapse.org/.

[231] Clark R.A., Shoaib M., Hewitt K.N., Stanford S.C., Bate S.T. (2012). A comparison of InVivoStat with other statistical software packages for analysis of data generated from animal experiments. J. Psychopharmacol..

[232] Du Sert N.P., Bamsey I., Bate S.T., Berdoy M., Clark R.A., Cuthill I.C., Fry D., Karp N.A., Macleod M., Moon L. (2017). The Experimental Design Assistant. Nat. Methods.

[233] Percie Du Sert N., Hurst V., Ahluwalia A., Alam S., Avey M.T., Baker M., Browne W.J., Clark A., Cuthill I.C., Dirnagl U. (2020). The ARRIVE guidelines 2.0: Updated guidelines for reporting animal research. J. Cereb. Blood Flow Metab..

[234] Wilkinson M.D., Dumontier M., Aalbersberg I.J., Appleton G., Axton M., Baak A., Blomberg N., Boiten J.-W., Da Silva Santos L.B., Bourne P.E. (2016). The FAIR Guiding Principles for scientific data management and stewardship. Sci. Data.

[235] Bains R.S., Huzard D., McCutcheon J.E., Boguszewski P., Virag D., Restivo L., Lewejohann L., Ashby M., Rozman J., Marion R. (2025). Too big to lose - a FAIR repository for biomedical data derived from home-cage monitoring. Open Science Framework.

